# A Multi-Modal Open Object Detection Model for Tomato Leaf Diseases with Strong Generalization Performance Using PDC-VLD

**DOI:** 10.34133/plantphenomics.0220

**Published:** 2024-08-13

**Authors:** Jinyang Li, Fengting Zhao, Hongmin Zhao, Guoxiong Zhou, Jiaxin Xu, Mingzhou Gao, Xin Li, Weisi Dai, Honliang Zhou, Yahui Hu, Mingfang He

**Affiliations:** ^1^ Central South University of Forestry and Technology, Changsha 410004, Hunan, China.; ^2^ Inner Mongolia Agriculture University, Hohhot 010010, Inner Mongolia Autonomous Region, China.; ^3^ Inner Mongolia University, Hohhot 010021, Inner Mongolia Autonomous Region, China.; ^4^ Plant Protection Institute, Hunan Academy of Agricultural Sciences, Changsha 410125, Hunan, China.

## Abstract

Precise disease detection is crucial in modern precision agriculture, especially in ensuring the health of tomato crops and enhancing agricultural productivity and product quality. Although most existing disease detection methods have helped growers identify tomato leaf diseases to some extent, these methods typically target fixed categories. When faced with new diseases, extensive and costly manual annotation is required to retrain the dataset. To overcome these limitations, this study proposes a multimodal model PDC-VLD based on the open-vocabulary object detection (OVD) technology within the VLDet framework, which can accurately identify new tomato leaf diseases without manual annotation by using only image–text pairs. First, we developed a progressive visual transformer-convolutional pyramid module (PVT-C) that effectively extracts tomato leaf disease features and optimizes anchor box positioning using the self-supervised learning algorithm DINO, suppressing interference from irrelevant backgrounds. Then, a context feature guided module (CFG) was adopted to address the low adaptability and recognition accuracy of the model in data-scarce environments. To validate the model’s effectiveness, we constructed a tomato leaf disease image dataset containing 4 base classes and 2 new categories. Experimental results show that the PDC-VLD model achieved 61.2% on the main evaluation metric ${\mathrm{mAP}}_{\text{novel}}^{50}$, and 56.4% on ${\mathrm{mAP}}_{\text{novel}}^{75}$, 87.7% on ${\mathrm{mAP}}_{\text{base}}^{50}$, 81.0% on ${\mathrm{mAP}}_{\mathrm{all}}^{50}$, and 45.5% on average recall, outperforming existing OVD models. Our research provides an innovative solution for efficiently and accurately detecting new diseases, substantially reducing the need for manual annotation, and offering critical technical support and practical reference for agricultural workers.

## Introduction

Tomatoes, as one of the major global crops, not only are popular with consumers due to their unique taste and rich nutritional value but also play a key role in the agricultural economies of many regions worldwide [1]. Unfortunately, like many crops, tomatoes are susceptible to various diseases and pests during their growth, substantially affecting yield and quality. It is estimated that global tomato production suffers millions of tons of losses due to pests and diseases each year, resulting in considerable economic damage [2]. Traditional methods of pest and disease detection—such as farmers’ empirical observations or expert guidance—are labor-intensive, inefficient, and prone to inaccuracies due to visual fatigue and individual experience biases [3,4].

**Fig. 1.**
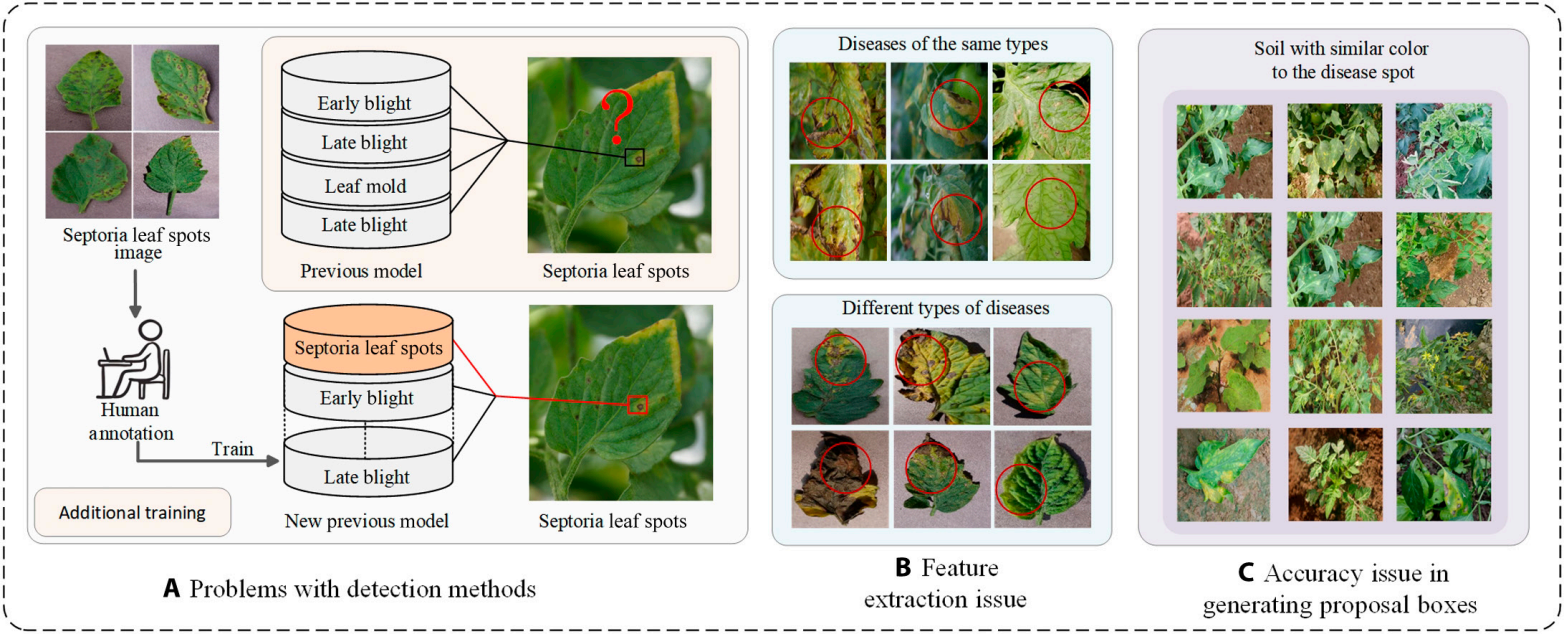
(A to C) The current issues with detection.

With technological advances, deep learning, as a potent technology within the field of artificial intelligence, has demonstrated its exceptional performance in numerous applications, including image processing and recognition [5–8]. Specifically, within the agricultural domain, deep learning has been applied to pest and disease identification [9–13],where it substantially improves the accuracy and efficiency of detection by learning from extensive data features. Nonetheless, as shown in Fig. 1A, current methods such as YOLO [14], Faster R-CNN [15], and other deep learning models, despite having achieved substantial results in accuracy and efficiency, still face challenges. These models rely heavily on large volumes of labeled data for training and are only capable of identifying disease types that are present in the training set [16]. When encountering unknown diseases, they often require additional data labeling and retraining [17], which is not only time-consuming and labor-intensive but also difficult to void bias during the annotation process. Therefore, reducing the dependence on extensive labeled data and enhancing the model’s rapid recognition capabilities for new types of tomato pests and diseases is crucial for improving detection efficiency and mitigating losses.

Over the past several years, researchers have extensively explored strategies to reduce the cost of data annotation [18–21]. In particular, open-vocabulary object detection (OVD) [22] has garnered substantial attention from both academic and industrial circles since its advent, owing to its capability to identify and localize an indefinite number of targets and categories without the need for manually expanding annotated datasets. It has brought new vitality and challenges to the conventional task of object detection and has achieved notable success. The concept of OVD was first proposed in the study of OVR-CNN [23], which employed a 2-stage training paradigm. In the first stage, the model leveraged image–caption pairs to pre-train the visual encoder paired with a multi-modal Transformer [24] to perform word mask prediction and enhance robustness. The second stage focused on training the detection model for base classes, which included bounding box regression and category matching. The model achieved category predictions by matching mixed image–text vectors obtained from the first stage with label word vectors. This 2-stage approach enabled the detection model to identify and locate targets of entirely new categories and has had a profound impact on subsequent OVD research. With burgeoning research in the field of large-scale cross-modal models, Region CLIP [25] leveraged CLIP [26], a powerful cross-modal large model, to train for the OVD task by mapping image regions to region-level descriptions to learn visual regional representation. This study employed CLIP to create pseudo-labels for region–text pairs and utilized contrastive loss to train the detection model before fine-tuning to elevate the performance of the visual encoder. Subsequent studies have progressed from various angles. HierKD [27] utilized a single-stage detector and introduced a module to distill global-level linguistic knowledge into visual representations, aiming to narrow the performance gap between single-stage and multi-stage approaches. This method used global knowledge distillation, aligning global image representations with corresponding caption embeddings through contrastive loss. PB-OVD [28] integrated Grad-CAM [29] with a region proposal network (RPN) to generate pseudo-bounding boxes derived from Grad-CAM activation maps, which matched pretrained visual-language model region embeddings with word embeddings. These pseudo-boxes were then utilized for joint training with existing annotated data. PromptDet [30] proposed a method of using category description prompts to explore category locations, enhancing the model’s recognition efficiency for new categories and recommended using online data augmentation to train new batches to boost learning effects. LocOV [31] used category-agnostic proposals in an RPN and trained Faster R-CNN by matching image regions with region features and word embeddings in captions. Detic [32] improved long-tail detection performance by supervising on the image level. OV-DETR [33] proposed a Transformer-based detector that addresses the OVD task through a conditional binary matching mechanism, providing a novel paradigm to improve the efficiency of language supervision. The OADP proposed by Wang et al. [34] pointed out that previous work only transferred object-level information from visual-language models to downstream detectors, neglecting the relationships between different objects. To address this issue, OADP adopted object-level distillation as well as global and chunk-level distillation strategies, aiming to bridge the relational information gap in object distillation by minimizing the global feature distance between the CLIP visual encoder and the detector backbone. VLDet [35] converted images and corresponding captions into sets of visual regions and text sequences and employed a set matching method to solve the visual-language alignment challenge. The research proposed a straightforward matching strategy to align captions with visual features, further advancing our understanding of this issue. It is evident that different teams tend to tackle the problem from various perspectives, exploring distinct strategies. As a hallmark of large-model research, OVD has indeed achieved substantial progress in reducing annotation work, bringing new vigor and challenges to traditional object detection tasks.

Upon a comprehensive analysis of the current state of open-vocabulary detection (OVD) technologies, we sought to investigate their applicability in the domain of tomato leaf disease recognition. Before embarking on our research, however, it was imperative to recognize the potential impact of environmental heterogeneity on disease detection [36,37]. Issues such as intraclass variability, where the same disease presents different morphologies, visual similarities between different diseases, and potential interference from environmental factors like soil backgrounds required careful consideration. We identified that the VLDet framework, capable of translating images and captions into regional and textual descriptions, offers a viable approach for extracting fine-grained information. This technology is essential for precise identification of tomato leaf diseases. As such, we were encouraged to explore its potential in new arenas. Our initial endeavors to deploy the VLDet framework for detecting tomato leaf diseases were met with several challenges that necessitated resolution: (a) Intra-class variation and inter-class similarity. As shown in Fig. 1B, even diseases of the same type may exhibit different colors, textures, and shapes at different stages of development or under different environmental conditions, leading to intra-class variability. Furthermore, diseases of different types may share similar colors, textures, morphologies, and other characteristics. These subtle variations make detection challenging. Traditional feature extraction methods often fail to capture all the key variations. These factors together result in inaccurate information extraction from images, thereby reducing the model’s performance in disease detection tasks. However, the networks like ResNet50, currently used by VLDet, often do not perform ideally in processing these complex features. Therefore, we need a feature extraction network that can better recognize complex disease characteristics. (b) The issue of interference from background. As demonstrated in Fig. 1C, the soil in the natural growth environment of tomatoes often presents a challenge in distinguishing it from certain diseases based on color characteristics, particularly when the disease occurs on the leaf margins. The interference from the soil can lead to misjudgments by the detection network. Additionally, variations in lighting conditions are also a factor that cannot be overlooked. Given the critical nature of the VLDet framework, accurate generation of candidate bounding boxes is of utmost importance for the subsequent object detection and matching processes. If the candidate boxes are not precise enough, they may overlook essential features or include irrelevant ones. This not only affects the accurate identification and localization of the disease but also leads to a waste of resources and adds extra challenges to the algorithm’s ability to accurately identify and locate the disease. The Faster-RCNN currently employed by VLDet tends to perform inadequately against such complex backgrounds, necessitating further optimization to enhance its performance in the face of these challenges. (c) Adaptive optimization: In scenarios where data on tomato leaf diseases is scarce, models may exhibit extreme behaviors in attempting to adapt or learn from these data. Such behaviors include slow convergence rates, suboptimal detection performance, a propensity for overfitting, and heightened sensitivity to random noise. These issues, in concert, complicate the achievement of efficient and accurate disease detection in practical applications while also escalating computational expenses.

Despite the substantial advancements of existing OVD models across various domains, their application to tomato leaf disease detection remains unexplored. Through a comprehensive analysis of the current state of OVD technology, we examined its applicability to tomato leaf disease identification. However, when applying the VLDet framework to tomato leaf disease detection, we encountered the following challenges: (a) Intra-class variation and inter-class similarity: As illustrated in Fig. 1B, diseases may exhibit different colors, textures, and shapes at various growth stages or under different environmental conditions, leading to intra-class variation. Additionally, different diseases may share similar features, complicating the detection process. Traditional feature extraction methods fail to accurately capture these variations, thereby reducing detection performance. The current VLDet framework, which utilizes ResNet50, performs poorly in handling these complex features, necessitating a more robust feature extraction network. (b) Background interference: As shown in Fig. 1C, the soil background in natural tomato growth environments often exhibits colors similar to those of diseases, especially when diseases occur at the edges of leaves. This interference can lead to misclassification by the detection network. The accurate generation of candidate bounding boxes is crucial for the object detection and matching process; inaccurate bounding boxes may overlook important features or include irrelevant ones, affecting disease recognition and localization. The currently used Faster-RCNN demonstrates insufficient performance in handling complex backgrounds and requires further optimization to enhance its capabilities. Additionally, variations in lighting conditions may exacerbate the background issues. (c) Adaptive optimization: In the context of scarce tomato leaf disease data, the model may exhibit extreme behaviors such as slow convergence, poor detection performance, overfitting, and high sensitivity to random noise. These issues impact the ability to perform efficient and accurate disease detection and increase computational overhead.

Recent studies provide us with perspectives on addressing certain challenges. Regarding (a) the issue of intra-class variation and inter-class similarity, Roy et al. [38] proposed an innovative real-time fine-grained object detection framework that effectively addresses multiple challenges in plant disease detection, including dense distribution, irregular morphology, multi-scale object categories, and structural similarity, by integrating DenseNet with an improved YOLOv4 algorithm. Deng et al. [39] introduced a multi-scale convolution module (MCM) structure, which utilizes convolution kernels of varying sizes to extract spatial information of tomato leaves at different scales, thereby resolving the issue of partial scale information loss inherent in traditional convolutions. Wang et al. [40] proposed a Transformer with a feature pyramid structure known as PVT, which can generate multi-scale feature maps similar to those from traditional CNN backbones [41], and can capture multi-scale, high-resolution outputs even with limited resources. Wang et al. [42] further improved upon PVT by introducing PVTv2, which utilizes zero-padding convolution [43] to achieve overlapping patch embedding and positional encoding with zero-padding, coupled with a linear complexity attention layer featuring mean pooling spatial-reduction attention (SRA)[44] . This allows PVTv2 to maintain more feature locality continuity and provides more flexibility in dealing with variable resolution images while maintaining a computational complexity akin to that of CNNs. For tomato leaf diseases with various pathological states, this CNN-integrated Transformer feature pyramid structure better learns multi-scale local features and global representations, accommodating a wealth of disease information, which enhances over the mere spatial down sampling of either feature maps or key/value matrices in PVT. Although PVTv2 excels at multi-scale feature extraction, it may still face the problem of feature confusion when dealing with plant leaf diseases that have a high degree of similarity. These advantages offer a reference for resolving feature extraction issues within networks. Regarding (b) the issue of interference from soil background, Roy and Bhaduri [45] proposed an improved YOLOv5 model, DenseSPH-YOLOv5, which incorporates DenseNet blocks and a Swin Transformer prediction head (SPH). This model integrates DenseNet blocks with the CSPDarknet53 backbone and introduces the convolutional block attention module (CBAM) and the SPH. By providing highly accurate local bounding box predictions, DenseSPH-YOLOv5 offers an effective and efficient model for damage localization, addressing the shortcomings of existing deep learning-based damage detection models. Although the DenseSPH-YOLOv5 model excels in damage localization, it may encounter issues with redundant predictions and difficulty in capturing subtle differences when dealing with scenes featuring dense spot characteristics. This is because DenseSPH-YOLOv5 may not be specifically optimized for dense spot features, leading to the generation of excessive bounding boxes during prediction or an inability to accurately distinguish similar spots. In contrast, Zhang et al. [46] presented an advanced end-to-end object detector known as DINO. DINO enhances performance and efficiency by using a denoising training approach based on contrastive methods, initiating anchors with a hybrid query selection method, and employing a dual forward scheme for box prediction. For characteristics with dense spots, this dual forward box prediction scheme effectively avoids redundant predictions and captures subtle differences between anchors, filtering out potential background noise elements. Concerning (c) the adaptation optimization issue, Hassani et al. [47] focused on the structural design for tiny datasets, including the utilization of small patch sizes, introducing convolutions in the shallow layers, and discarding classification tokens. However, the use of small patch sizes leads to a quadratic increase in computational complexity, which is often unacceptable in practical applications. Li et al. [48] suggested a strategy to augment the performance of vision transformers (VTs) [49] on small-scale datasets by integrating features from lightweight CNNs as local guidance. This method leverages the feature representation of advanced CNN models as a starting point to aid VT in effective learning for tasks in small datasets. Combining CNN as an auxiliary learning pathway without compromising VTs’ independent learning capacity plays to VTs’ strength in modeling complex data relationships. Its core value lies in mitigating the difficulties VT faces when learning from small datasets, thereby accelerating model convergence and improving final performance. In this study, we draw on this methodology, introducing a locality guidance strategy to boost the learning efficiency of the model’s image encoding for small datasets.

In accordance with the methodologies of prior research, we have tackled the challenge of accurately extracting complex features of tomato leaf diseases (problem A) by incorporating convolutional projections into a pyramid-structured VT. This integration harnesses the hierarchical characteristics of the VT along with the local sensitivity of convolutional projections, thereby augmenting the model’s ability to capture both local and global spatial contexts; convolutional projection can better capture local features and perform effective transformations in the feature space, thereby enhancing the model’s ability to distinguish fine differences. To efficiently suppress irrelevant background interference elements in images (problem B), we have applied the DINO technology to the realm of tomato leaf diseases, thereby enhancing the precision of anchor box calibration. Finally, to address the extreme behaviors manifested by the network under conditions of dataset scarcity (problem C), we have introduced a context feature guided module (CFG) module to guide the Transformer network in more effectively learning and capturing hierarchical information.

This paper mainly makes the following contributions:

1. Construction and preprocessing of the tomato disease dataset: In order to train our network model, we constructed an image dataset containing 6,923 instances of tomato leaf diseases. We meticulously preprocessed the dataset and performed careful annotation, marking text descriptions and precise bounding boxes for all disease areas to ensure that the model can learn effective recognition patterns from the data.

2. We proposed a multimodal OVD model for tomato leaf diseases, PDC-VLD, which exhibits robust generalization capabilities and can accurately identify new types of tomato leaf diseases without the need for manual annotation.

a. For the first time, OVD technology is applied to tomato leaf disease detection. Utilizing the VLDet framework, this approach learns directly from image–text pair data, aligning objects with language by matching a set of image region features with a set of word embeddings, enabling the identification of unknown diseases.

b. We propose the progressive visual transformer-convolutional pyramid module (PVT-C), which combines the hierarchical properties of pyramid-structured VTs with the local sensitivity of convolutional projections. This enhancement strengthens the model’s ability to capture both local and global spatial contexts, thereby more effectively extracting fine-grained disease-related features.

c. The VLDet framework incorporates a novel tomato leaf disease anchor box calibration strategy based on DINO. DINO’s dual forward prediction scheme and hybrid query selection mechanism reduce redundant predictions and effectively remove distracting background elements from the images, improving the accuracy and reliability of the final predictions.

d. We employ a CNN-based local feature guidance (CFG) method for adaptive optimization of tomato leaf diseases. This method, first applied within the VLDet framework, leverages the CFG module to guide the transformer network in more effectively learning and capturing hierarchical information. This simplifies the knowledge transfer and network training processes, substantially enhancing the performance of leaf disease detection.

3. The extensive experiments conducted on the self-built tomato leaf disease dataset using PDC-VLD demonstrate that we have improved ${\mathrm{mAP}}_{\text{novel}}^{75}$, ${\mathrm{mAP}}_{\text{novel}}^{50}$, ${\mathrm{mAP}}_{\text{base}}^{50}$, ${\mathrm{mAP}}_{\mathrm{all}}^{50},$ and average recall (AR) by 11.9%, 10.2%, 15.8%, 14.4%, and 7.4%, respectively, outperforming other methods. This method can directly learn from image–text pairs to recognize and detect tomato leaf diseases and can identify some unseen disease types. To our knowledge, this is the first time the work of OVD has been applied to the detection of tomato leaves. This phase of work is not only a new attempt to test the capabilities of the VLDet framework but also a bold experiment in upgrading and innovating traditional agricultural technology, bringing new possibilities to OVD and related fields.

## Methods

### Dataset

**Fig. 2.**
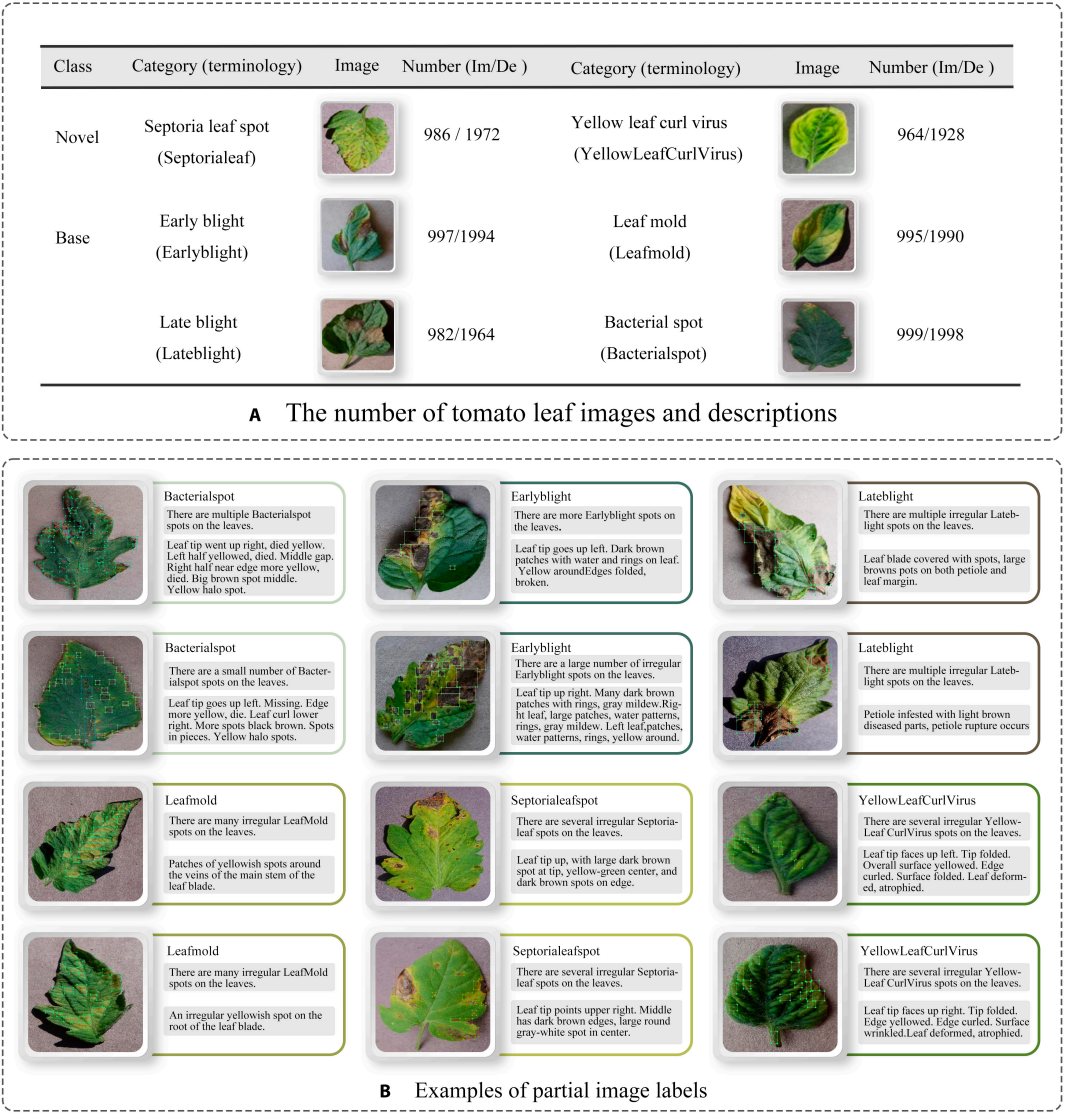
(A and B) Examples of partial image labels.

The dataset in this paper is primarily composed of 2 parts. One part comes from Plant Village [50], and the other part consists of images taken with a Canon camera at the Plant Protection Research Institute of the Hunan Academy of Agricultural Sciences in Changsha City, Hunan Province, in March 2023. The dataset includes a total of 6,923 images, both of 6 disease samples and healthy samples. The diseases include bacterial spot, early blight, late blight, leaf mold, septoria leaf spot, and yellow leaf curl virus. To better meet the needs of our study, we first categorized the 6 disease classes into 4 basic categories (bacterial spot, early blight, late blight, leaf mold) and 2 new categories (septoria leaf spot and yellow leaf curl virus) and then processed all images to a uniform size of 255 × 255 pixels. Subsequently, we manually annotated them using the Labelme software. Additionally, we sought the expertise of professionals from the Agricultural Academy of Sciences in Changsha City, Hunan Province, China, to provide textual descriptions for the content of 6,923 images. This process took 120 days. We provided detailed annotations for the content of each tomato leaf disease image, specifically divided into titles and content descriptions. Figure 2A shows all the components of our dataset, which includes 6,923 images, 6,923 annotation files, and 13,864 text annotations. The information for each annotated image is stored in COCO.JSON format.

It merits emphasis that, in response to the specialized exigencies of tomato leaf disease research, this study discerned a shortfall in conventional word vector repositories with regard to their coverage of relevant technical terminologies. To ameliorate this deficit, we retrained the pertinent vocabulary representations. The employment of pre-trained word embeddings for precise noun segmentation constitutes an essential foundational task in textual data processing. This task not only is crucial for the accurate establishment of alignment between image regions and textual lexicons but also exerts a profound impact on the precision of the entire research framework. For instance, in the compound noun phrase “yellow leaf curl virus,” adjectives or nominal adjectives such as “yellow,” “leaf,” and “curl” collectively modify the core noun “virus,” rendering it a term that encapsulates a singular concept. The nomenclature of such multi-word compositions of diseases presents considerable challenges to standard word vector training. Although existing embedding algorithms can recognize some standard noun phrases, their efficacy diminishes when confronted with elongated, domain-specific noun phrases, thereby escalating complexity. The inability to accurately classify such compound nouns as unified entities may adversely affect the efficacy of subsequent target detection tasks. To diminish the risk of error propagation during the word vector training phase, this study engineered a bespoke terminological dataset for 6 prevalent tomato foliar diseases, implementing a novel naming strategy by amalgamating multiple words into a new term, thereby reducing their lengths, without encountering any specific amalgamation requisites. Subsequently, employing the Bert model [51], we successfully trained a series of pre-trained word embeddings specifically tailored for this terminological set. In the “Experiments on the rationality of dataset design” section, the efficacy of this methodology was experimentally validated, ensuring the accuracy and reliability of the research findings.

Figure 2B presents detailed annotations of images of 6 types of bacterial leaf blight, intricately depicting the unique distribution of lesions and damage sustained by the leaves. We observed that both early blight and late blight cause brown spots on tomato leaves, with spots sharing similar texture patterns, providing a rich source of information for training the network to capture precise characteristics of the lesions. Bacterial spot disease forms dense clusters of small spots along the leaf margins; these clear patterns enable the network to distinguish between infected areas of the leaf and similar background disturbances, and accurately identify the distribution of the disease. Simultaneously, the pale-yellow spots caused by downy mildew enrich the network’s diagnostic data dimension, facilitating its ability to differentiate between yellow spots caused by disease and other nonspecific yellowing areas.

These comprehensive descriptions of disease symptom features are essential for the understanding of patterns by deep learning networks and for mastering the characteristic pale-yellow lesion features of downy mildew, which also empowers the network to precisely capture the key visual clues of these diseases. This rich feature knowledge base provides a solid foundation for the model in broad disease classification, enhancing the model’s ability to recognize emerging diseases in practical applications. Therefore, we choose to conduct in-depth research on these tomato leaf diseases.

### PDC-VLD

**Fig. 3.**
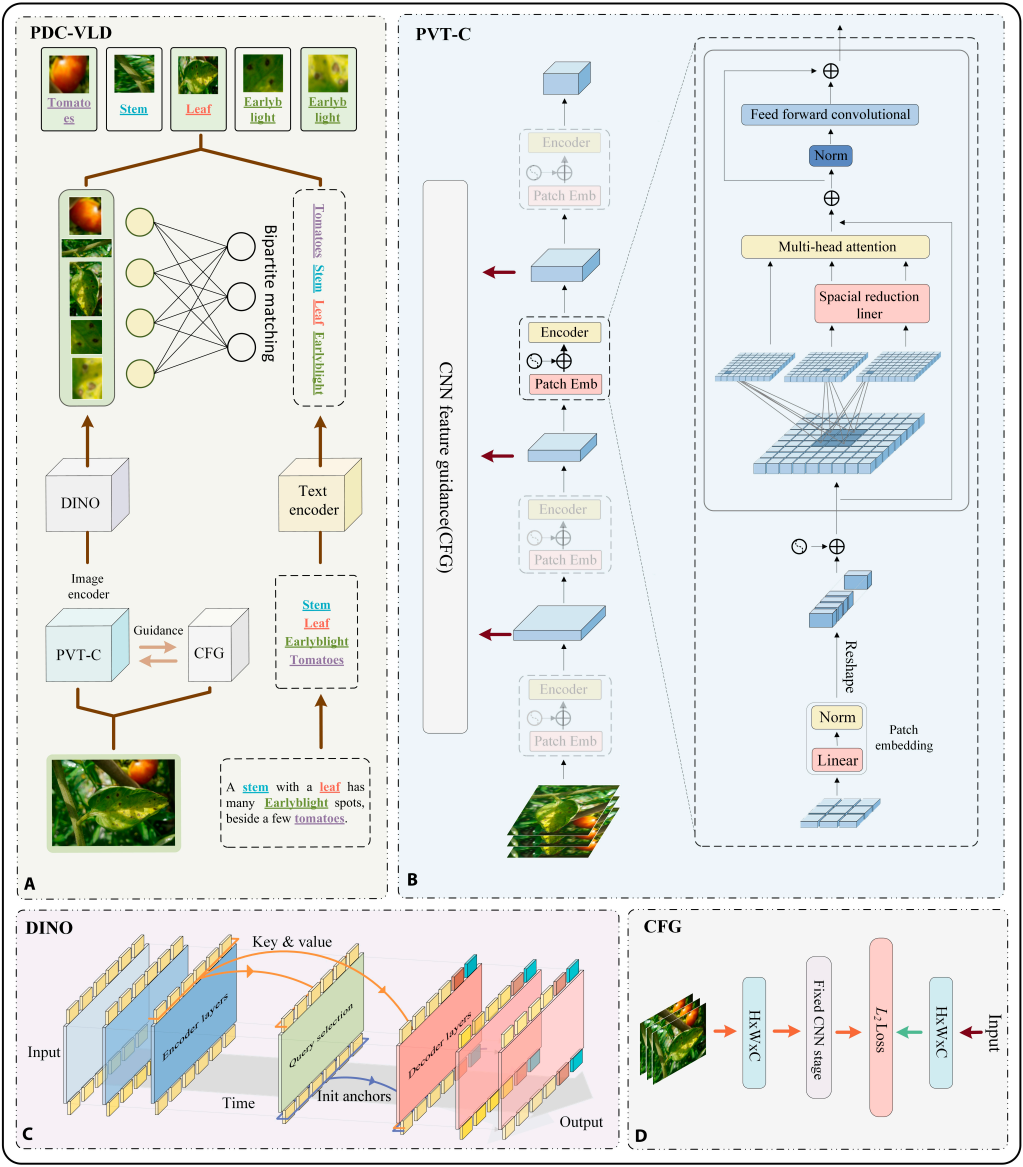
(A to D) PDC-VLD structure diagram.

To enable efficient and accurate detection of new diseases without the need for manual labeling, we propose an open-vocabulary target detection network for tomato leaf disease detection named PDC-VLD, with its specific structure shown in Fig. 3A. This network includes 3 main components: a visual object detector, a text encoder, and a region-to-word matching alignment module. For the visual object detector, we propose a new PVT-C feature extraction module to extract effective features from the input image sequences. These features are passed to the designed DINO end-to-end object detector to generate prediction boxes. During the feature extraction phase, CFG is used to guide pre-trained knowledge into the model, promoting rapid convergence and enhancing detection performance. On the textual side, we train word embeddings to capture the semantics and contextual relations between the words in our training dataset. During the training process, we use a bipartite graph matching to achieve precise alignment between image region features and corresponding word embeddings. In Fig. 3E, we detail the specific processes of the steps outlined above. Initially, the image is fed into both PVT-C and CFG simultaneously. Guided by CFG, PVT-C extracts feature from the image, which is then processed by DINO to generate features of the image regions. On the textual side, nouns from the sentence are extracted and transformed into text features by a text encoder. Last, a bipartite graph matching is utilized to precisely align the image region features with the corresponding word embeddings. In Table 1, we present the detailed configurations of PVT-C and DINO, with CFG utilizing the Resnet-56 model. The “VLDet” section will introduce the architecture of the VLDet framework, the “PVT-C” section will detail the design of PVT-C, the “DINO” section will elaborate on the details of the DINO architecture, and the “CFG” section will focus on explaining the implementation and benefits of the CNN local guidance strategy.

**Table 1.** Detailed settings of PDC-VLD series

Method		Output size	Layer name	
PVT-C	Stage 1	$\frac{H}{4}\times \frac{W}{4}$	Overlapping patch embedding	*S*_1_ = 4,*C*_1_= 64
			Transformer encoder	*Conv. Proj.* = 3 × 3,192 *R*_1_ = 8,*N*_1_ = 1,*E*_1_ = 4,*L*_1_ = 3
	Stage 2	$\frac{H}{8}\times \frac{W}{8}$	Overlapping patch embedding	*S*_1_ = 2,*C*_1_ = 128
			Transformer encoder	* Conv. Proj.* = 3 × 3,128 *R*_1_ = 4,*N*_1_ = 2,*E*_1_ = 4,*L*_1_ = 6
	Stage 3	$\frac{H}{16}\times \frac{W\;}{16}$	Overlapping patch embedding	*S*_1_ = 2,*C*_1_ = 320
			Transformer encoder	* Conv. Proj. *= 3 × 3,320 *R*_1_ = 2,*N*_1_ = 5,*E*_1_ = 4,*L*_1_ = 40
	Stage 4	$\frac{H}{32}\times \frac{W}{32}$	Overlapping patch embedding	*S*_1_ = 2,*C*_1_ = 512
			Transformer encoder	* Conv. Proj.* = 3 × 3,512 *R*_1_ = 1,*N*_1_ = 8,*E*_1_ = 4,*L*_1_ = 3
DINO	Stage 1	$\frac{H}{4}\times \frac{W}{4}$	Overlapping patch embedding	*S*_1_ = 4,*C*_1_ = 64
			Transformer encoder	*N*_1_ = 1,*D*_1_ = 64 *R*_1_ = 4,*L*_1_ = 2
	Stage 2	$\frac{H}{8}\times \frac{W}{8}$	Overlapping patch embedding	*S*_1_=2,*C*_1_=128
			Transformer encoder	*N*_1_ = 2,*D*_1_ = 128 *R*_1_ = 4,*L*_1_ = 4
	Stage 3	$\frac{H}{16}\times \frac{W}{16}$	Overlapping patch embedding	*S*_1_ = 2,*C*_1_ = 256
			Transformer encoder	*N*_1_ = 4,*D*_1_ = 256 *E*_1_ = 4,*L*_1_ = 8
	Stage 4	$\frac{H}{32}\times \frac{W}{32}$	Overlapping patch embedding	*S*_1_ = 2,*C*_1_ = 512
			Transformer encoder	*N*_1_ = 8,*D*_1_ = 512 *R*_1_ = 4,*L*_1_ = 16
	Head	1 ×1	1,000	
Params			171.17M	
FLOPs			114.05G	
FPS			32	

Among them, *S*_*i*_ is the stride of the overlapping patch embedding in stage i, *C*_*i*_ is the channel number of the output of stage i, *L*_*i*_ is the number of encoder layers in stage i, *R*_*i*_ is the reduction ratio of the SRA in stage i, *P*_*i*_ is the adaptive average pooling size of the linear SRA in stage i, *N*_*i*_ is the head number of the efficient self-attention in stage i, *E*_*i*_ is the expansion ratio of the feed-forward layer in stage i, *D*_*i*_ is the embedding dimension in stage i, and *Conv. Proj* is convolutional projection.

#### VLDet

To achieve detection of new diseases without the need for manual labeling, we apply the VLDet framework to the field of tomato leaf disease recognition. This framework encompasses 3 main components, and here, we primarily introduce its unique region-to-word matching alignment module.

This framework leverages a corpus of image–text pairs to learn fine-grained region-word alignment, which is constructed as a set-to-set matching problem, where a set of image region candidates is paired with a set of word candidates. We formulate this region-word alignment as an optimization problem of bipartite graph matching, rather than generating pseudo-bounding boxes and labels for each image. For a given image *I*, the image encoder produces a series of candidate region features *R = [r*_1_*, r*_2_*,..., rm]*, where m denotes the number of regions under consideration. For a caption *C*, we extract all nouns from the caption and embed each noun using a language encoder. The resulting word embeddings are *W = [w*_1_*, w*_2_*,...,w*_|w|_*]*, with *|W|* being the number of nouns in the caption. Generally, the number of regions m is greater than the number of nouns *|W|*, since the RPN provides a wide range of candidate regions. We redefine the task of region-to-word mapping from a global perspective, transforming it into a many-to-many bipartite graph matching problem. The cost between image regions and words is determined by alignment scores *S = WR^T^*. This bipartite graph matching problem can then be efficiently resolved by using the well-established Hungarian algorithm [52]. After matching, the classifier head of the detection model is optimized by calculating the cross-entropy loss, which is represented as follows:

$$L_{\text{region}-\text{word}}=\sum_{i=1}^{|W|}-\left[\text{logσ}(s_{ik})+\sum_{j\in W^{\prime }}\log(1-\sigma (s_{jk}))\right]$$ (1)

Here, σ represents the sigmoid activation function, *s*_*ik*_ represents the alignment score between the *i*th noun embedding and the *k*th regional feature that matches it in the bipartite graph matching, and W^’^ is the set of nouns in the same batch of captions that have not been matched with any region. In this formula, by calculating the loss of matched and unmatched noun–region pairs, the correct matches are strengthened while the incorrect ones are penalized.

After transforming images into multiple regions and segmenting text into words, a bipartite graph matching algorithm is used to extract associations between regions and words at a global level. This method allows us, for new categories, to train object detection models with free supervision conducted through image–text pairs, without relying on any specific object category language annotations.

#### PVT-C

The detection of diseases on tomato leaves faces multiple challenges, including the visual similarity between different diseases, the variation in the appearance of the same disease under different conditions, and the accurate identification of diseases against complex backgrounds. Existing CNN-based methods excel in extracting local features, but the lack of global information may limit CNN performance when it comes to capturing diffused disease patterns on leaves or discerning subtle differences between the background and diseases. Meanwhile, although VTs can effectively integrate long-distance features through their self-attention mechanism, they often overlook the detailed local information. Therefore, there arises a need to integrate the local sensitivity of CNNs with the global awareness of VTs without adding an excessive computational burden.

As mentioned in Introduction, Wang et al. [42] demonstrated the potential of utilizing convolution to enhance local feature extraction and the fusion of global information through the Transformer’s self-attention mechanism. Inspired by these prior studies, this research designs the PVT-C module, aimed at specifically addressing the challenges in detecting diseases on tomato leaves. As described in Fig. 3C, PVT-C employs multi-level outputs to acquire feature maps of different resolutions while increasing the convolutional projection to strengthen feature extraction efficiency. By properly adjusting the size of feature mappings and combining them with Transformer encoders that have removed positional embeddings, PVT-C optimizes the balance between local and global features during the recognition process. Moreover, we introduce a new type of depthwise separable convolutional projection [53], which enhances the model’s ability to recognize diseases near the edges, thus ensuring detection accuracy while reducing computational complexity.

By adjusting scales at each stage, a feature pyramid can be constructed for the Transformer. To build a feature pyramid and optimize the resolution of feature mappings, our process is similar to PVT2. Starting with the input image size of *H* × *W ×* 3, we first divide it into $\frac{\mathrm{HW}}{4^{2}}$ patches of size 4 × 4 × 3 in the first stage. After flattening these patches, they are projected into a linear space to produce embeddings of dimension $\frac{\mathrm{HW}}{4^{2}}$ *× C*_1_, which are then combined with positional embeddings, passed through L_1_ layers of Transformer encoders, and reconstructed into feature mappings *F*_1_ of size $\frac{H}{4}\;$ *×* $\frac{W}{4}$ *× C*_1_. Subsequently, the same method is utilized to progressively raise the pyramid level and generate subsequent feature mappings *F*_2_, *F*_3_, and *F*_4_, which have strides of 8, 16, and 32 pixels, respectively. Considering the importance of high-resolution features, we restrict the stride of the highest resolution mapping to 4. To adjust the scale of feature mappings at each stage, assuming P_i_ is the block size at stage i, we uniformly divide the previous stage’s feature mapping into $\begin{array}{c} F_{i-1}\in R^{H_{i-1}\;\times \;W_{i-1}\;\times \;C_{i-1}}\;\text{into}\;\frac{H_{i-1}\;\times \;W_{i-1}}{P_{i}^{2}} \end{array}$ blocks, then flatten these blocks, and project them into a C-dimensional embedding space. After linear projection, the size of the embedded patches becomes $\frac{H_{i-1}}{P_{i}}\times \frac{W_{i-1}}{P_{i}}\times C_{i}$, with the height and width of the input reduced by a factor of *P_i_*, respectively.

Transformer encoder at stage i has an encoder layer, and each encoder layer is composed of an attention layer and a feedforward layer [37]. Similar to the SRA used in PVT2, our attention layer receives a query Q, a key K, and a value V as inputs and uses average pooling to reduce the spatial dimensions (*h × w*) to a fixed size before the attention operation and then outputs a refined feature. The difference is that we removed positional embeddings, and the linear projections before each self-attention block in the transformer module are replaced by the convolutional projections proposed by us, which further enhances the model’s ability to capture local features.

More specifically, tokens are first reshaped into 2D token maps. Next, a depthwise separable convolution with a kernel size of 5 is used to implement the convolutional projection. Finally, the projected tokens are flattened into 1D for subsequent processing. This is represented as follows:

$$x_{i}^{q/k/v}=\text{Flatten}\left(\text{Conv}2d\left(\text{Reshape}2D\left(x_{i}\right),s\right)\right)$$ (2)

$x_{i}^{q/k/v}\;$is the token input for the Q/K/V matrices of the *i*th layer, *x_i_* represents the undisturbed tokens before the convolutional projection, and Conv2d is a depthwise separable convolution, where s denotes the size of the convolutional kernel.

By constructing the aforementioned pyramid structure and applying depthwise separable convolutions, the integration of local and global information has been effectively balanced, making it more suitable for precise extraction of disease characteristics. Experiments related to PVT-C can be found in the “Effectiveness of PVT-C” section.

#### DINO

The VLDet framework generates candidate bounding boxes through an object detection network and combines them with a bipartite matching algorithm to link these candidate boxes to the corresponding words. Therefore, the focus of this framework is on accurate location detection, with less importance placed on classification compared to traditional object detection tasks. In this context, there are strict requirements on the quality of the candidate boxes: (a) Coverage: Candidate boxes need to extensively cover all potential text areas to ensure that no critical information is missed during the matching process. (b) Precision: Avoid mistakenly including background areas within candidate boxes to reduce the risk of mismatching. (c) Minimize false positives: Reduce the cases of mistakenly marking non-text areas as candidate boxes in order to avoid adding complexity to subsequent processing.

As mentioned in Introduction, the DINO model proposed by Zhang et al. [46] is highly referential. We have integrated DINO into the VLDet framework for the first time to enhance its detection capabilities in specific contexts such as tomato leaf disease detection. We made adaptive adjustments to DINO by removing the classification component while retaining the rest of the structure, which includes a multi-layer Transformer encoder and decoder, as can be seen in Fig. 3E. This structural modification allows the model to focus on generating high-quality candidate bounding boxes that are relevant to text matching, making it suitable for use within the VLDet framework. First, the image is fed into the PVT-C network to extract multi-scale feature maps. These feature maps are then immediately followed by a series of positional embeddings to endow each feature with spatial information, ensuring awareness of their spatial positions in subsequent processing. After the position encoding treatment, these features are sent to the Transformer encoder, where they are enhanced through self-attention and feed-forward network layer operations. Afterward, a unique mixed query selection strategy is employed, which uses anchor boxes as the starting point for position queries in the decoder while maintaining a learnable content query mechanism, enabling the model to adapt to complex scenes and target morphologies through iterative processes. Once the anchor initialization and content queries are prepared, our model relies on deformable attention to process the features output by the encoder, allowing the model to more flexibly focus on key areas of the target object. This process involves multiple rounds of layer-by-layer updates to refine the predictions for the anchor boxes at each step. In addition, the model includes a dedicated denoising branch that uses a contrastive denoising training strategy to handle difficult or misleading samples, enhancing the model’s resilience in the face of noise and interference. Furthermore, a novel forward twice-training method is adopted, which optimizes learning between adjacent layers through gradient propagation, ensuring benefits from refined anchor box information while also improving the performance of earlier layers.

In summary, the DINO model effectively addresses the 3 key issues of coverage, accuracy, and minimizing false positives through the following methods. First, DINO employs a contrastive denoising training strategy, enabling the model to accurately identify and distinguish text regions even in samples containing noisy backgrounds, thereby extensively covering all potential text regions. This ensures that no critical information is missed during the matching process. Second, in terms of accuracy, DINO enhances the precise localization of text regions by introducing refined positional encoding and deformable attention mechanisms. These mechanisms allow the model to better focus on key features, preventing the inclusion of background regions within the candidate bounding boxes. Additionally, the model’s dual forward prediction mechanism allows for self-correction after initial predictions, gradually adjusting the position and size of the bounding boxes to reduce matching errors, thus ensuring that the bounding boxes tightly enclose the actual text regions. Finally, in minimizing false positives, DINO combines contrastive denoising training with a hybrid query selection strategy, enhancing the model’s ability to filter out irrelevant information by training it to recognize and ignore noisy backgrounds. Specialized denoising branches and multiple iterative update mechanisms further assist the model in effectively distinguishing actual text regions from background noise, substantially reducing false positives and avoiding added complexity in subsequent processing. Through these optimization measures, the DINO model ensures that the generated candidate bounding boxes are predominantly valid text regions, thereby improving the overall performance and reliability of the system.

By adapting DINO and integrating it into the VLDet framework, we can effectively reduce the generation of duplicate boxes and erroneous boxes, thereby improving the accuracy of detection. Experiments related to DINO can be found in the “Effectiveness of DINO” section.

#### CFG

It is difficult to train transformer networks from scratch on small datasets using normal training strategies [54–56], as they lack hierarchical information from the local to the global, which can lead to some extreme situations. In order to train more effectively and efficiently, it is necessary for us to design special adapter modules to enable normal training. Inspired by the work of Li et al. [48], who proposed a method of guiding VTs with lightweight CNN features, which can effectively improve model performance on small datasets, we first trained a lightweight CNN model on the dataset.

In Fig. 3D, we present the results of the lightweight CNN trained on the dataset. The training comprises 2 main tasks: first is to minimize the semantic gap between the token sequences generated by the layers of PVT-C and the CNN features. This is accomplished by calibrating the features in both the spatial and channel dimensions, incorporating the distance between features as an optimizable loss function into the training process. Second, under the guidance of class labels, PVT-C is able to learn independently and build its own understanding of the image content. Our goal is to efficiently train PVT-C in a data-limited context, so the modifications are focused on the training strategy, without the need for additional changes to the structure of PVT-C. It is worth noting that during inference, the CNN model is no longer relied upon. For an input image *X* ∈ *R*^*H* × *W* × 3^, the information flow of PVT-C can be represented as follows:

$$T_{0}=\text{PatchEmbedding}\left(X\right),T_{0}\in R^{L\times C}$$ (3)

$$T_{i}=\text{Block}\left(T_{i-1}\right),T_{i}\in R^{L\times C}$$ (4)

In the formula, *T*_0_ represents the initial token sequence generated by the image, *T_i_* represents the token sequence after processing by the *i*th transformer encoder block, *L* represents the number of tokens, and *C* represents the dimension of the embedding space. CNNs typically contain multiple processing stages, and as the model depth increases, the resolution of the feature maps gradually decreases. The information processing flow of a CNN can be characterized as follows:

$$M_{1}={\text{Stage}}_{1}\left(X\right),M_{1}\in R^{H_{1}\times W_{1}\times C_{1}}$$ (5)

$$M_{i}={\text{Stage}}_{i}\left(M_{i-1}\right),M_{i}\in R^{H_{i}\times W_{i}\times C_{i}}$$ (6)

where *M_i_* is the feature map after stage *i*. Given the token sequence from PVT-C denoted as *T_i_* ∈ *R*^*L* × *C*^ and feature maps from CNN denoted as $M_{i}\in R^{H_{i}\times W_{i}\times C_{i}}$, there is a mismatch in their spatial and channel dimensions, and to facilitate the optimization process, it is first necessary to map these features into the same dimensional space. Initially, the visual output of PVT-C is reshaped to restore the spatial structure of the feature map. This operation is based on the intrinsic 2-dimensional nature of images in space. Subsequently, to ensure accuracy in the calculation of the distance metric between features, we synchronize the adjustment of these 2 sets of features so that they match in size, namely, the maximum feasible width and height of the feature maps. This adjustment involves a linear up-sampling strategy. The mathematical description of this series of spatial feature alignment operations is as follows:

$${\hat{T}}_{i}=\text{Reshape}\left(T_{i}\right),{\hat{T}}_{i}\in R^{H_{t}\times W_{t}\times C}$$ (7)

$$\hat{H}=\text{max}\left(H_{t},H_{j}\right),\hat{W}=\text{max}\left(W_{t},W_{j}\right)$$ (8)

$${\hat{F}}_{i}=\text{Resize}\left(T_{i}\right),{\hat{F}}_{pvt-c}\in R^{\hat{H}\times \hat{W}\times C}$$ (9)

$$F_{cnn}=\text{Resize}\left(M_{j}\right),F_{cnn}\in R^{\hat{H}\times \hat{W}\times C_{j}}$$ (10)

where *F*_*pvt* − *c*_ and *F_cnn_* respectively represent the spatial transformation features of PVT-C and CNN. To align the channel dimensions, learnable point-wise linear projection is applied to the features from PVT-C, with specific operations as follows:

$$F_{pvt-c}=\text{Linear}\left({\hat{F}}_{pvt-c}\right),F_{pvt-c}\in R^{\hat{H}\times \hat{W}\times C_{j}}$$ (11)

where Linear refers to a learnable linear projection, realized through a 1 × 1 convolution.

Regarding the details of guidance, with the 2 one-to-one sets of transformed features {$F_{\mathrm{pvt}-c}^{i}$*| i = 1,2, ···, k*} and {$F_{\mathrm{cnn}}^{j}$*| j = 1,2, ···, k*} we use L_2_ distance metric to realize feature guidance:

$$L_{\text{guidance}}=\sum_{i=1}^{k}\frac{1}{{\hat{H}}_{i}\times {\hat{W}}_{i}}{||F_{pvt-c}^{i}-F_{cnn}^{j}||}_{F}^{2}$$ (12)

where *k* is the number of features chosen to perform guidance. The total loss can be expressed as follows:

$$L=L_{cls}+\beta L_{\text{guidance}}$$ (13)

Among these, *L_cls_* is the cross-entropy loss for the classification task. The final loss consists of 2 parts that correspond to the 2 tasks, where *L_cls_* allows PVT-C to learn independently, and *L_guidance_* forces PVT-C to imitate the features learned by the CNN to better integrate local information. With this dual-task setup, PVT-C has the capacity to express in its own manner, rather than just copying the features learned by the CNN. The hyperparameter *β* is used to balance between imitation and self-learning.

Overall, this approach cleverly integrates CNNs with Transformers, realizing the complementary strengths of both architectures. Such a training regimen not only alleviates the model’s propensity for overfitting on small datasets but also accelerates the convergence speed, which is particularly precious for application scenarios with smaller datasets of tomato leaf diseases like ours. Through this unique training mode, the performance of PVT-C on the tomato leaf disease dataset has been substantially enhanced. For experiments related to CFG, please refer to the “Effectiveness of CFG” section.

## Results and Discussion

This section demonstrates through experimentation that PDC-VLD has overcome the challenges of applying the VLDet framework to the detection of tomato leaf diseases, thereby validating the superiority of the model. The subsections are organized as follows: The “Experiment setting and implementation details” section describes the experimental setup; the “Evaluation indicators” section evaluates the experimental metrics of PDC-VLD; the “Module effectiveness experiment” section analyzes the performance of individual modules within PDC-VLD, confirming the effectiveness of our proposed method; the “Ablation experiment” section conducts ablation experiments on PDC-VLD to verify its effectiveness; the “Comparison of visualization results” section visualizes the detection results of PDC-VLD to assess its capabilities intuitively; the “Experiment comparing PDC-VLD with other models” section compares PDC-VLD with other deep learning network models to demonstrate its advantages in the task of tomato leaf disease detection; the “Generalization experiments” section evaluates the generalization capability of PDC-VLD; the “Rationality experiment of dataset design” section analyzes the impact of dataset design on PDC-VLD to demonstrate the rationality of our dataset design. The “Real-world experiments” section evaluates the real-world performance of PDC-VLD.

### Experiment setting and implementation details

In order to ensure the reliability of the PDC-VLD experimental results and to eliminate interference caused by variations in the experimental environment, all experiments in this study were conducted on the same hardware and software platform. The core of the hardware configuration for this platform was provided by the AutoDL platform. In terms of software, we used a unified development environment, which included specific versions of the operating system and a suite of software tools, to maintain the consistency of the experiments. A detailed list of the hardware configurations and software experimental setups is presented in Table 2.

**Table 2.** Hardware and software parameters

Hardware environment	CPU	12 vCPU Intel(R) Xeon(R) Platinum 8255C CPU @ 2.50 GHz
	GPU	NVIDIA Tesla V100-SXM2
	RAM	300 GB
	Video memory	32 GB
Software environment	OS	Ubuntu 20.04.4
	CUDA Toolkit	V11.3
	CUDNN	V8.0.4
	Pytorch	1.11.0

The learning rate was increased from 0 to 0.002 over the first 200 iterations. The model was trained using a stochastic gradient descent (SGD) optimizer with a batch size of 4 for 10,000 iterations. The learning rate was decreased by a factor of 10 at both the 7,000 iterations and 9,000 iterations. For the dataset, image data augmentation was performed prior to input, including symmetrical flipping, adding Gaussian noise, altering brightness and shadows, and textual annotations that were expanded, resulting in a total of 14,657 images and 47,992 texts. A set of 11,725 images annotated with bounding boxes for base classes was used as the training set, and a set of 1,466 images annotated with bounding boxes for both base and novel classes was employed as the validation set. Additionally, a set of 1,466 images annotated with bounding boxes for both base and novel classes was used as the test set.

### Evaluation indicators

In this study, we use the AR and the mean average precision (mAP) at thresholds of 0.5 and 0.75 to evaluate the performance of the detection model.

There are 2 commonly used indexes precision (P) and recall (R). P denotes the proportion of correct classification, and R denotes the proportion of relevant information detected to the total. They can be defined as follows:

$$P=\frac{TP}{TP+FP}$$ (14)

$$R=\frac{TP}{TP+FN}$$ (15)

To evaluate the method’s ability to detect novel classes, we divide the average precision (AP) metric into 2 types: *AP_novel_* and *AP_base_*. Our primary focus is the measurement of *AP_novel_*.The mAP is calculated using the following formula:

$$mAP=\int_{0}^{1}P\left(R\right)dR$$ (16)

The AR is primarily used to assess the degree of model detection failure. The formula for calculating AR is as follows:

$$AR=\frac{\text{Recall}}{n}$$ (17)

where *n* is the number of detected image frames.

### Module effectiveness experiment

This section provides a detailed analysis of the evaluation metrics and parameters for PVT-C, DINO, and CFG. The effectiveness of each innovative module has been verified under the same experimental setup. “Basic” refers to the benchmark network VLDet, which utilizes Faster R-CNN as the framework for object detection and ResNet50 [41] as the backbone network for feature extraction. An exhibition of the experimental results is presented below.

#### Effectiveness of PVT-C

In our study, we introduce a novel feature extraction module named PVT-C, designed to extract features from images more effectively and enhance the performance of the model. To validate the effectiveness of PVT-C, we conducted experiments and documented the results in detail in Table 3. The object detector we utilized was the Faster R-CNN. During these experiments, we compared PVT-C with some of the current leading feature extractors. The experimental data signify that PVT-C achieves performance improvement on key performance metrics for OVD. Compared to other models, PVT-C not only is adept at distinguishing between different disease types with similar visual features but also can precisely identify variations in individual diseases under different environmental conditions and accurately locate and identify diseases against cluttered backgrounds. Through this enhanced feature extraction mechanism, PVT-C substantially improves the model’s accuracy in identifying new types of diseases in complex environments, demonstrating its potential and prospects as an image feature extractor.

**Table 3.** Module effectiveness experiment

Model	Method	${\mathrm{mAP}}_{\text{novel}}^{75}$	${\mathrm{mAP}}_{\text{novel}}^{50}$	${\mathrm{mAP}}_{\text{base}}^{50}$	${\mathrm{mAP}}_{\mathrm{all}}^{50}$	AR
Basic	ResNet50 [41]+ FR-RCNN [15]	44.5	51.0	71.9	66.6	38.1
PVT-C	ResNet50[41]	44.5	51.0	71.9	66.6	38.1
	PAC-CSPDarknet-53 [36]	44.1	51.4	72.6	67.4	38.0
	CSPDarkNet [55]	45.1	51.8	73.9	68.1	38.1
	T2T-24[56]	45.5	52.4	74.5	68.9	38.3
	PVT-Large [40]	45.6	53.3	76.2	70.4	38.4
	TNT-B [58]	46.3	54.1	77.8	71.8	38.7
	CvT-21 [59]	46.1	54.0	77.5	71.6	38.5
	Swin-B [60]	47.0	54.9	78.6	72.6	38.9
	Twins-SVT-L [61]	47.7	55.1	78.9	72.9	38.9
	PVT2-B5 [42]	48.1	55.3	79.3	73.3	39.2
	PVT-C	48.8	55.7	80.0	73.9	39.5
DINO	Faster-RCNN [15]	44.5	51.0	71.9	66.6	38.1
	DETR (DC5) [62]	21.5	32.3	48.3	44.3	22.3
	Dynamic DETR (5scale) [63]	44.4	51.2	72.5	67.1	38.3
	Dynamic Head (5scale) [64]	44.8	51.9	73.8	68.3	38.8
	HTC (5scale) [65]	45.5	52.4	74.7	66.5	38.2
	DN-De-DETR (5scale) [66]	46.3	53.1	76.0	70.2	39.2
	DINO-5scale	50.7	55.1	79.6	73.4	41.0
CFG	Rn56 + FR-RCNN	28.1	40.1	52.4	49.3	20.8
	ResNet50 + FR-RCNN	44.5	51.0	71.9	66.6	38.1
	RN50 + FR-RCNN + Guidance	47.1	54.3	77.2	71.4	39.0
	Swin-B + FR-RCNN	49.7	54.9	78.6	72.6	40.6
	Swin-B + FR-RCNN + Guidance	53.3	58.4	85.1	78.4	44.0
	PVT-C + DINO	52.9	57.6	81.3	75.3	41.9
	PVT-C + DINO + Guidance	56.4	61.2	87.7	81.0	45.5

#### Effectiveness of DINO

In the object detection module of this study, we introduced the DINO model for the first time to explore its capability to identify targets within complex background scenes, as well as its potential to reduce false positives. To thoroughly evaluate the efficacy of DINO, we conducted a comprehensive comparison with current leading object detection algorithms. The standard ResNet50 network was selected as the backbone for feature extraction. As demonstrated by the results shown in Table 3, DINO exhibited a substantial performance improvement in the accuracy of tomato leaf disease detection, which is attributed to the synergistic effect of several key factors: First, DINO reduced the occurrence of false positives, especially during target localization; second, thanks to DINO’s unique dual-prediction mechanism and denoising training strategy based on contrastive learning, it enhanced the clarity of target edge region recognition and also displayed remarkable denoising capabilities in data environments with high levels of noise. These enhancements contributed collectively to its superior detection performance.

#### Effectiveness of CFG

For the optimization of the network training process, we propose a module called CFG, whose purpose is to guide the learning process of deep learning models. In order to verify the effectiveness of the CFG module, a series of experiments were conducted in this study, as shown in Table 3, where Resnet-56 was used as the guiding model and as a baseline for comparison (group 1 experiments).

The experimental results show that the introduction of the CFG module improves the performance of various network structures. This is due to the fact that the CFG module contains a large amount of local knowledge, which allows the Transformer module to learn more meaningful and generalizable information and process the hierarchical information more efficiently, thus improving the ability to capture the characteristics of tomato leaf disease infestation.

In order to validate the design options in our approach, we investigated the complexity of the hyperparametric *β*, CNN models used to balance imitation and self-learning. All results given are based on PVT-C + DINO(PCD).

To verify the effect of the hyperparameter *β*, we use different *β* uniformly distributed in [0,3.0]. As shown in Table 4 experimental results, the *β* coefficients play a key role in the trade-off between imitation and self-learning. Low values of *β* weaken the strength of the imitation learning signals. When the *β* coefficients are adjusted within a moderate range, CFG can substantially optimize the performance.

**Table 4.** Compare different factor *β* and guidance models

* β*	${\mathrm{mAP}}_{\text{novel}}^{50}$	${\mathrm{mAP}}_{\text{base}}^{50}$		${\mathrm{mAP}}_{\mathrm{all}}^{50}$
0.0	57.6	81.3		75.3
0.5	57.9	81.6		75.6
1.0	58.4	82.2		76.2
1.5	59.1	83.1		77.0
2.0	60.3	84.6		78.5
2.5	61.2	87.7		81.0
3.0	60.9	85.4		79.2
Method	CNN ${\mathrm{mAP}}_{\text{novel}}^{50}$ - 28.6 40.1 46.3			PCD ${\mathrm{mAP}}_{\text{novel}}^{50}$57.6 59.5 61.2 61.1	
None					
Resnet-20					
Resnet-56					
Resnet-110					

For the guidance model, we use 3 CNNs with the same structure but different number of layers. For comparison, we only consider *mAP_novel_* here. From Table 4, we can find that although these 3 CNNs show a huge performance gap, the difference between the VT improvements they bring is relatively small. This phenomenon suggests that our method only guides the VTs to learn localization rather than completely transferring the knowledge of the CNN, which makes our method very efficient by using lightweight CNNs.

### Ablation experiment

In order to test the effectiveness of our VLDet-based approach, we performed ablation experiments on the PDC-VLD dataset, and the specific experimental results are displayed in Table 5. Through the control variable method, we introduced 3 modules, PVT-V, DINO, and CFG, one by one, and executed 8 sets of exhaustive ablation tests in conjunction with the VLDet framework. The experimental results show that the CFG module substantially improves the ${\mathrm{mAP}}_{\text{novel}}^{50}$ baseline performance with an average increase of 5.5%, while PVT-C and DINO each bring 4.7% and 4.1% accuracy improvement to ${\mathrm{mAP}}_{\text{novel}}^{50}$. Combining the above results, it can be concluded that all 3 components, PVT-C, DINO, and CFG, have a positive impact on the model’s mAP, which verifies that our proposed improvements are effective. Therefore, PDC-VLD outperforms the original VLDet model on the tomato leaf disease detection task.

**Table 5.** Ablation experiment

PVT-C	DINO	CFG	${\mathrm{mAP}}_{\text{novel}}^{75}$	${\mathrm{mAP}}_{\text{novel}}^{50}$	${\mathrm{mAP}}_{\text{base}}^{50}$	${\mathrm{mAP}}_{\mathrm{all}}^{50}$	AR
			44.5	51.0	71.9	66.6	38.1
√			48.8	55.7	80.0	73.9	39.5
	√		50.7	55.1	79.6	73.4	41.0
		√	49.8	56.5	80.7	74.6	40.7
√	√		52.9	57.6	81.3	75.3	41.9
√		√	54.6	59.8	83.5	77.5	42.5
	√	√	53.2	58.6	82.2	76.3	43.0
√	√	√	56.4	61.2	87.7	81.0	45.5

### Comparison of visualization results

Table 6 shows a visual comparison of our disease detection results using VLDet and PDC-VLD to provide a deeper understanding of the performance of PDC-VLD. For the sake of observational comparison, we limit the presented results to the recognized output of disease names. The 6 groups A to F presented correspond to the following diseases: bacterial spot, early blight, leaf mold, late blight, septoria leaf spot, and yellow leaf curl virus. To better showcase the results, we will only present the outcomes related to disease here.

**Table 6.** Comparison of visualization test results

Class	Method				Group
	VLDet	+PVT-C	+DINO	PDC-VLD	
Base	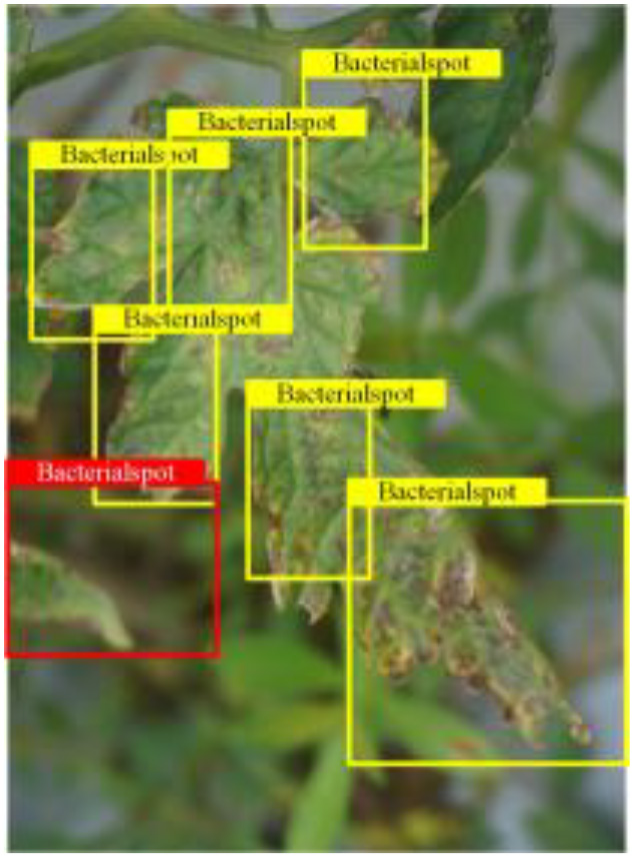	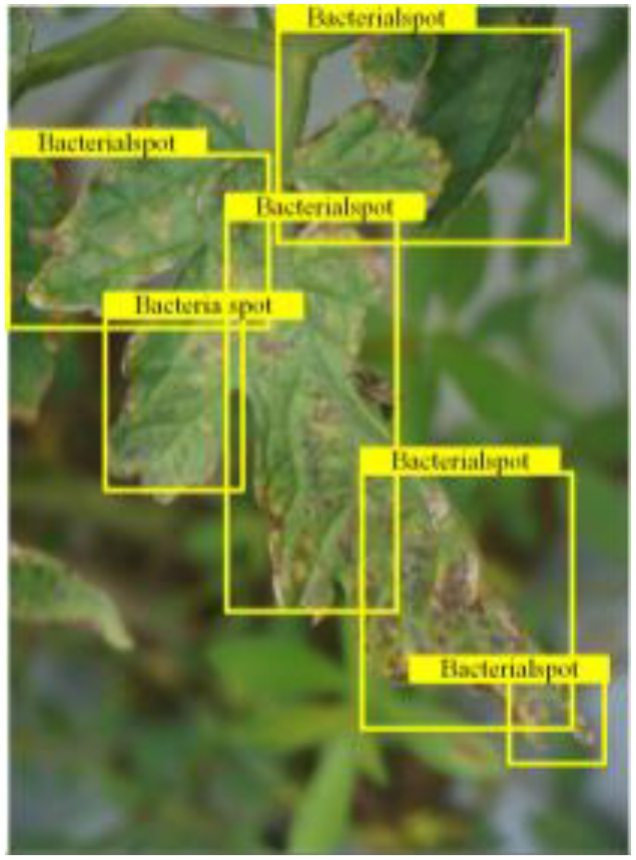	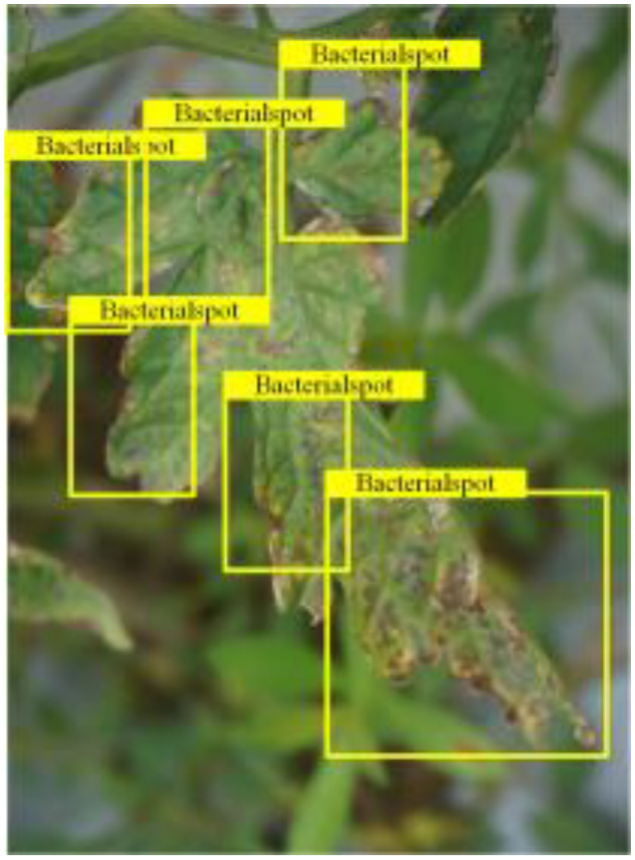	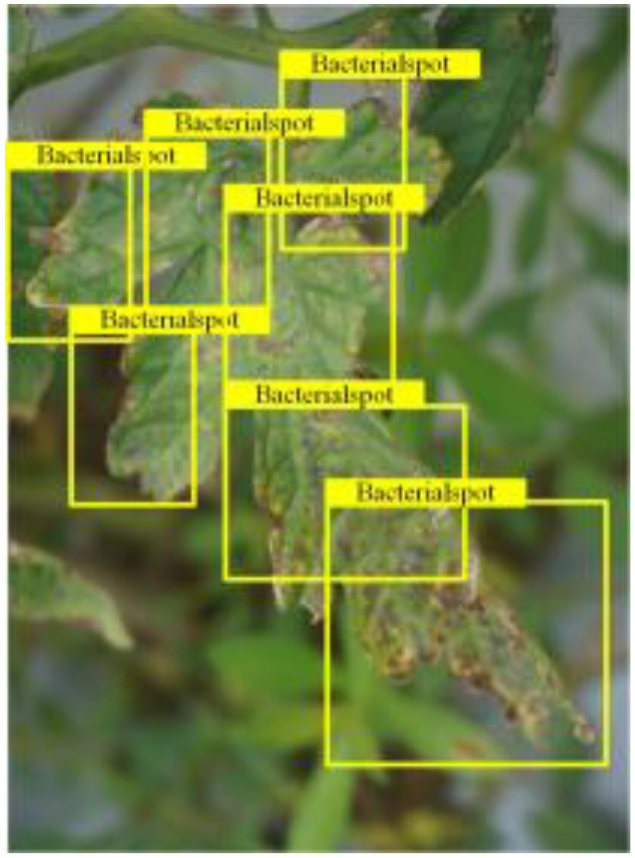	A
	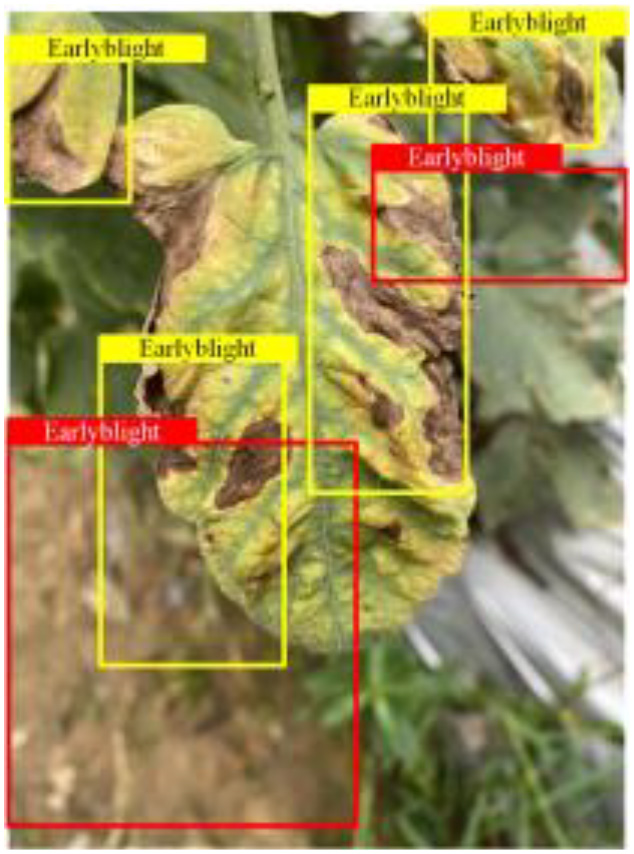	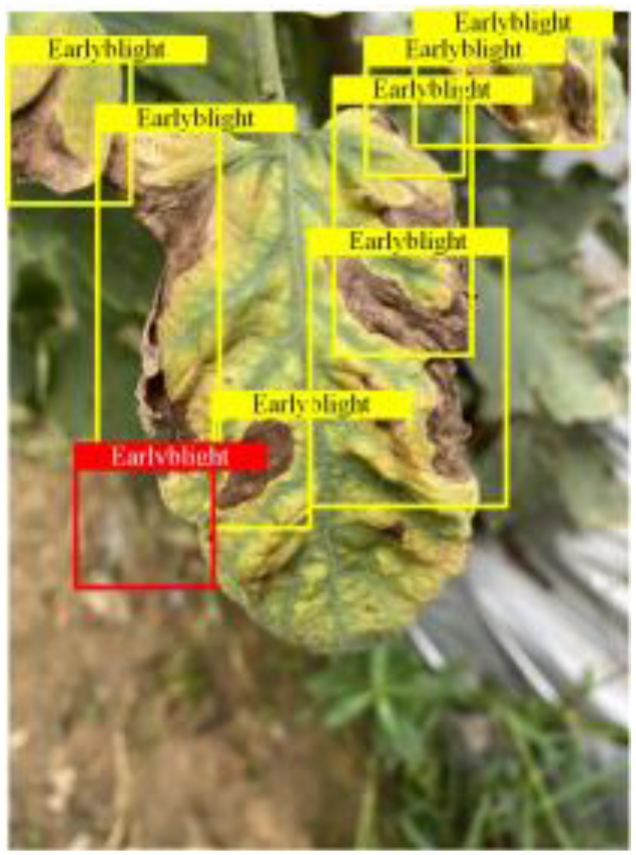	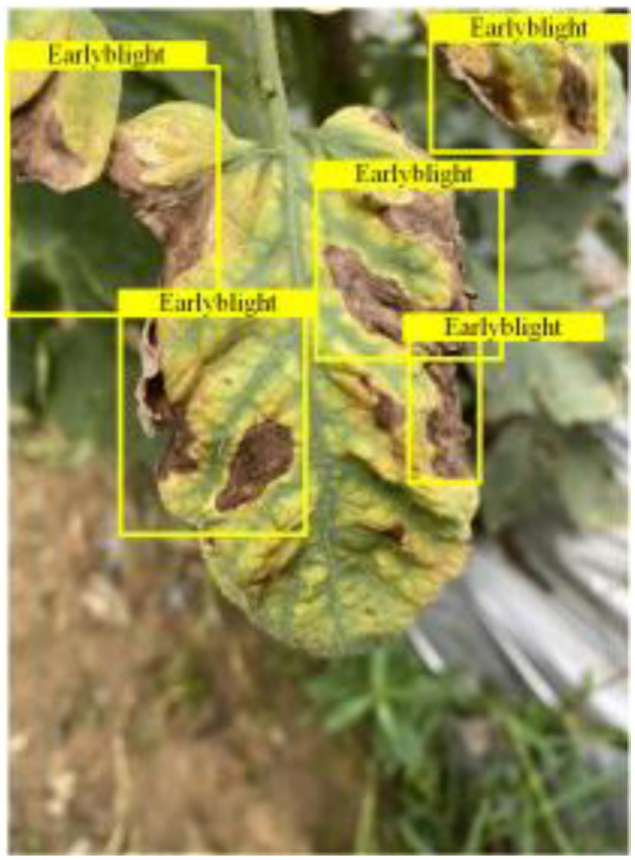	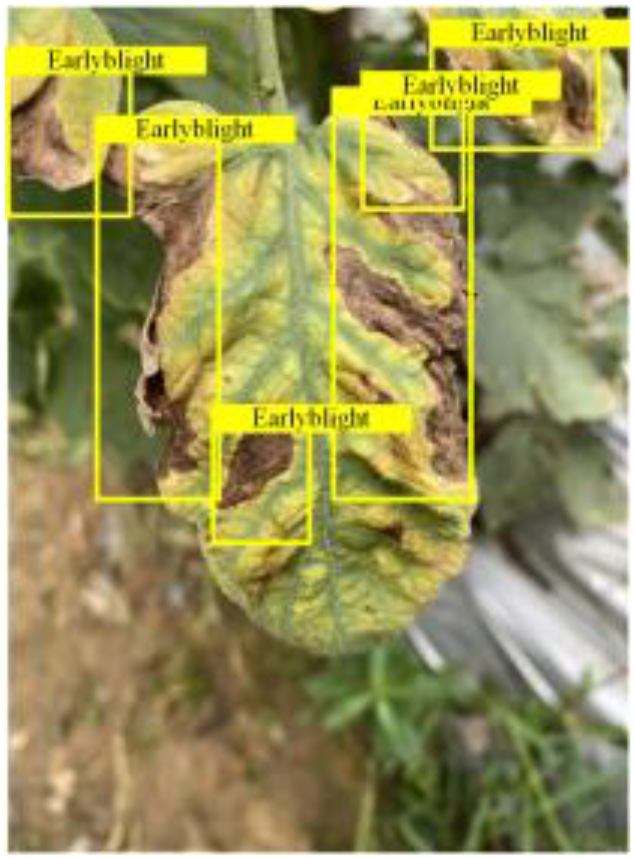	B
	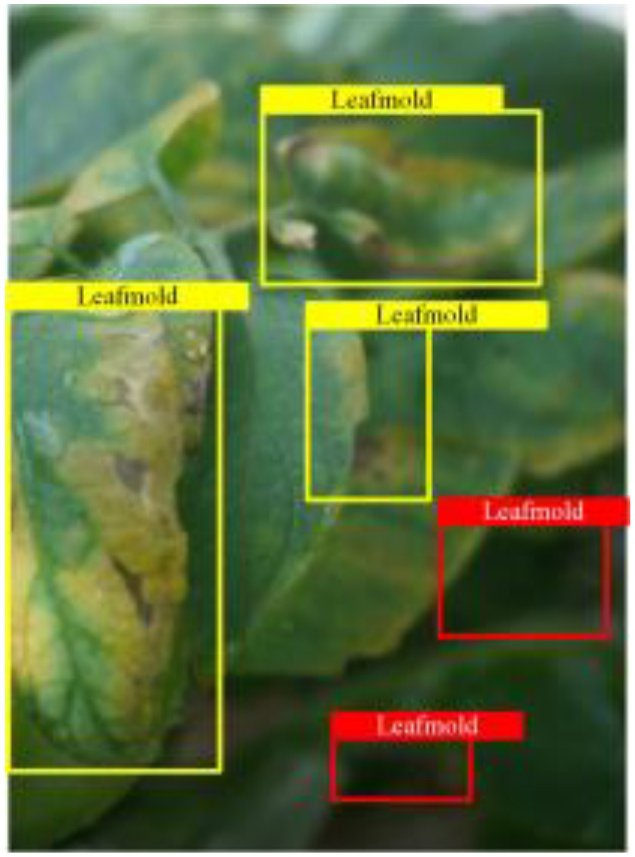	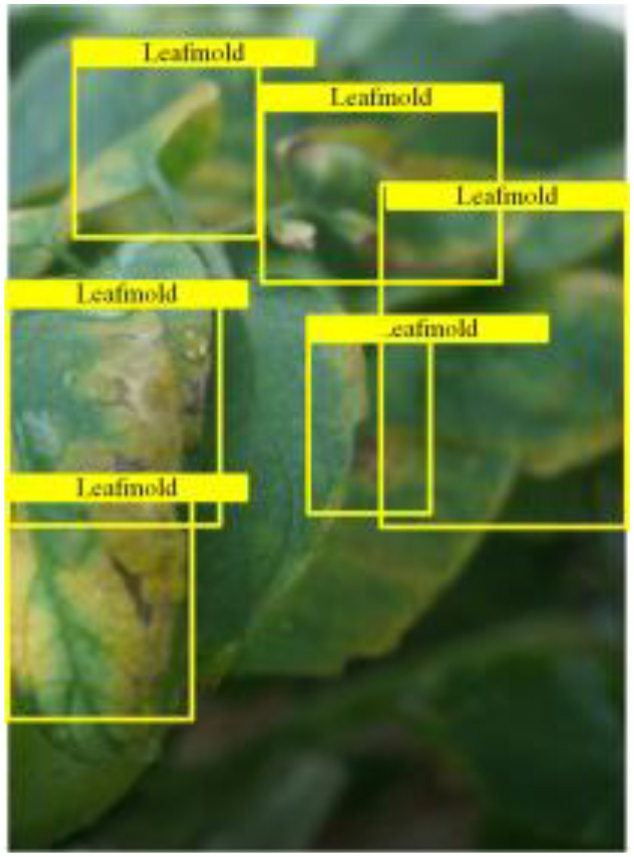	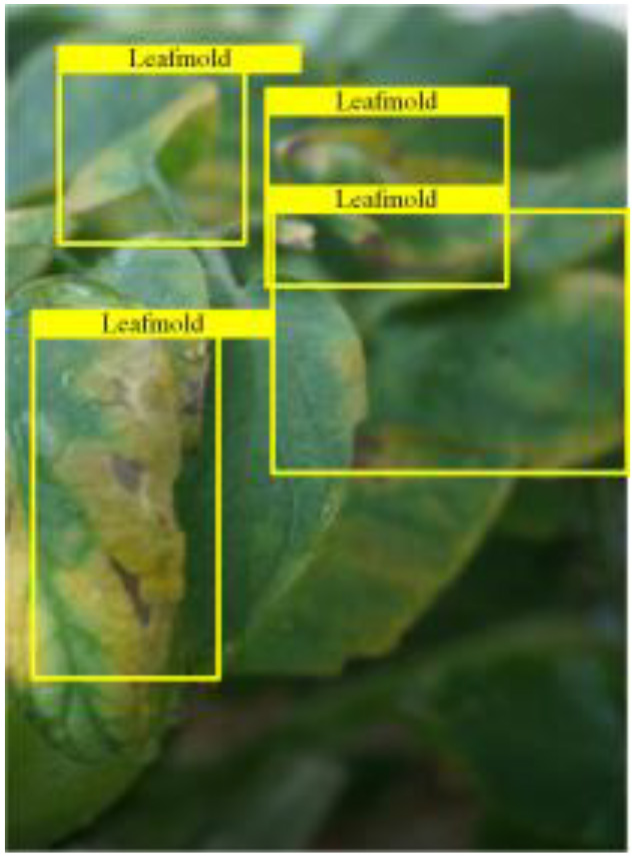	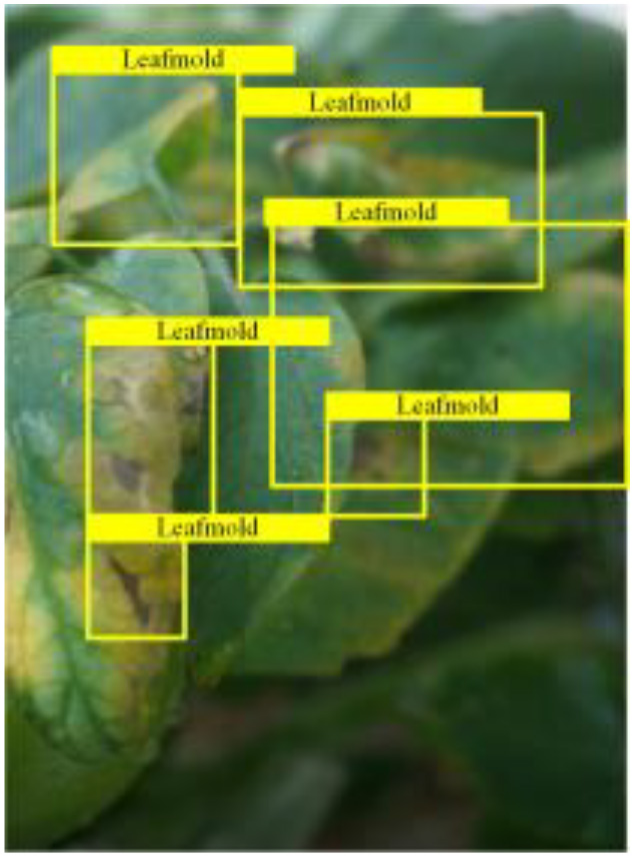	C
	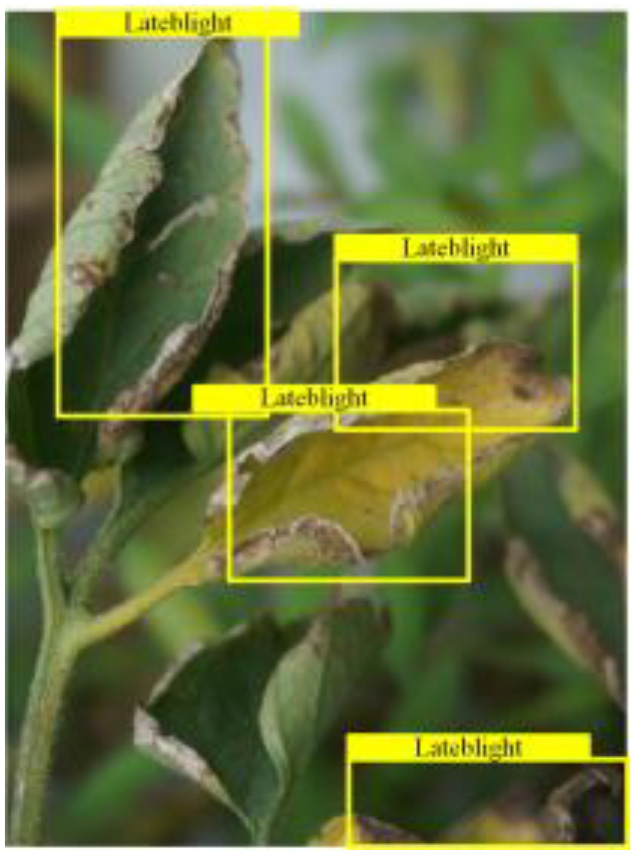	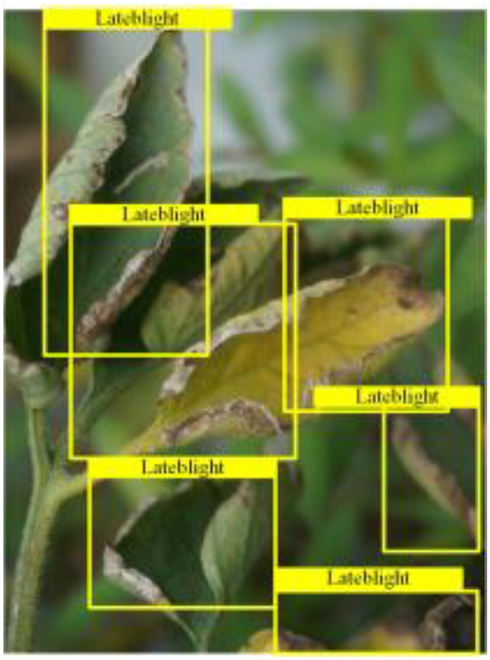	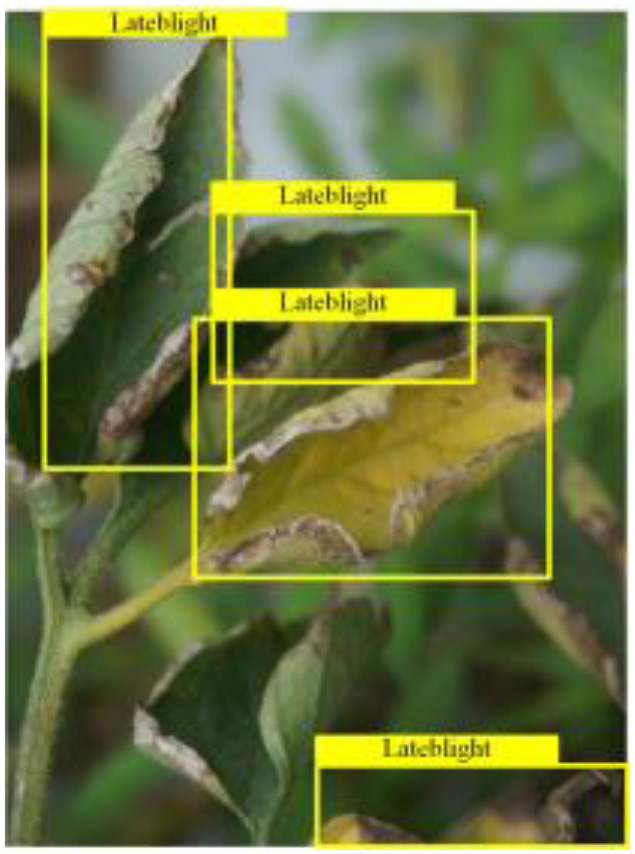	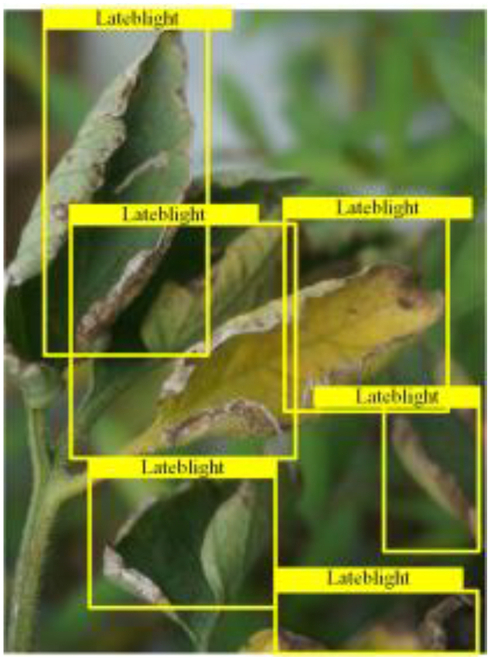	D
Novel	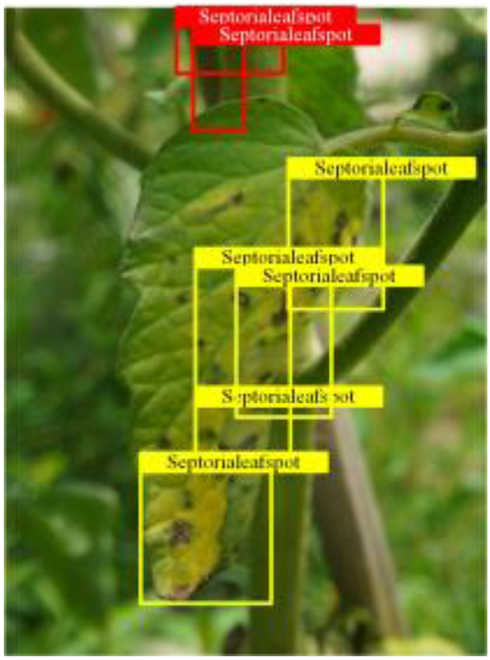	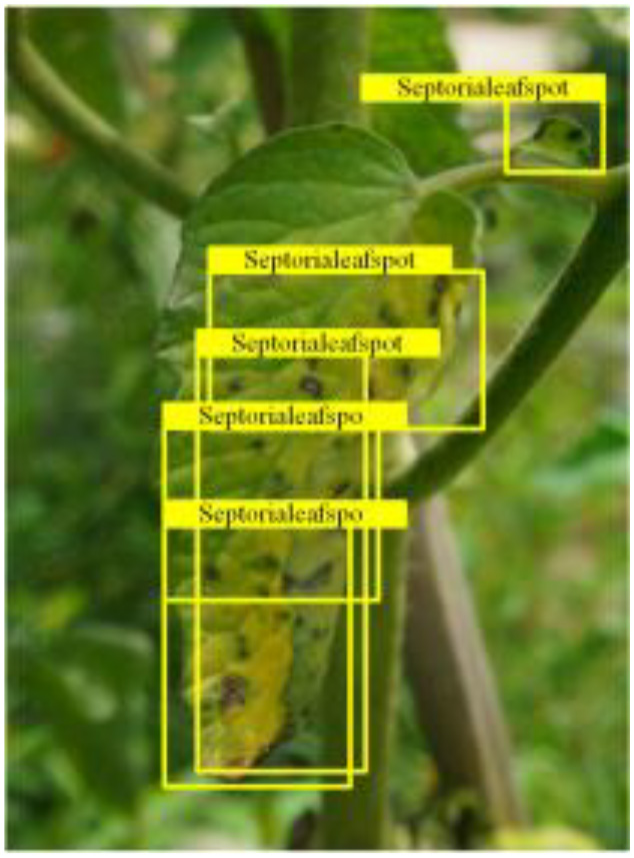	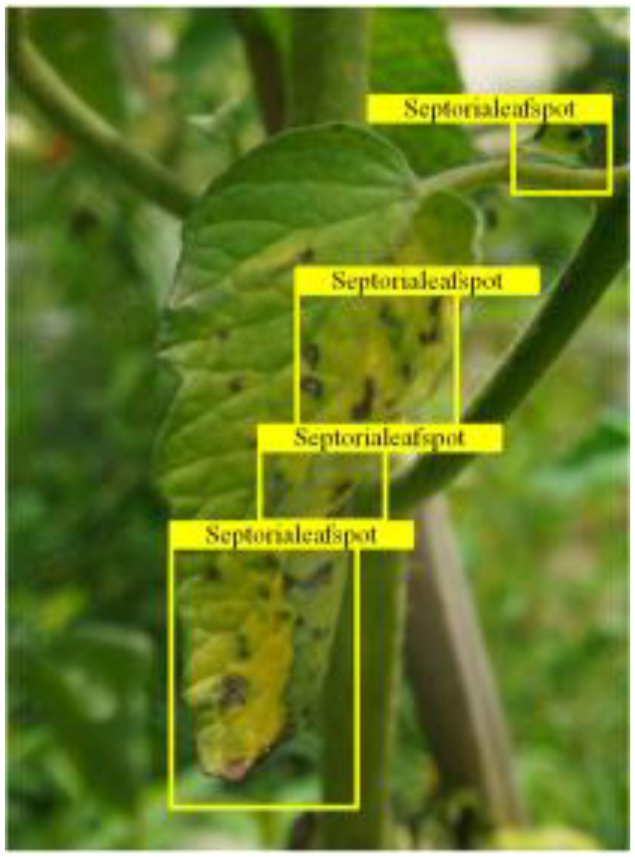	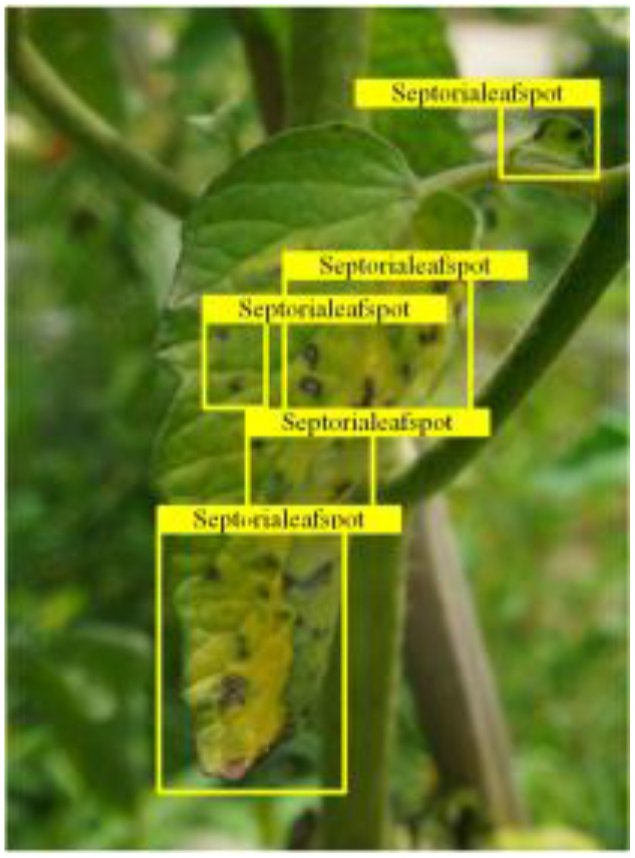	E
	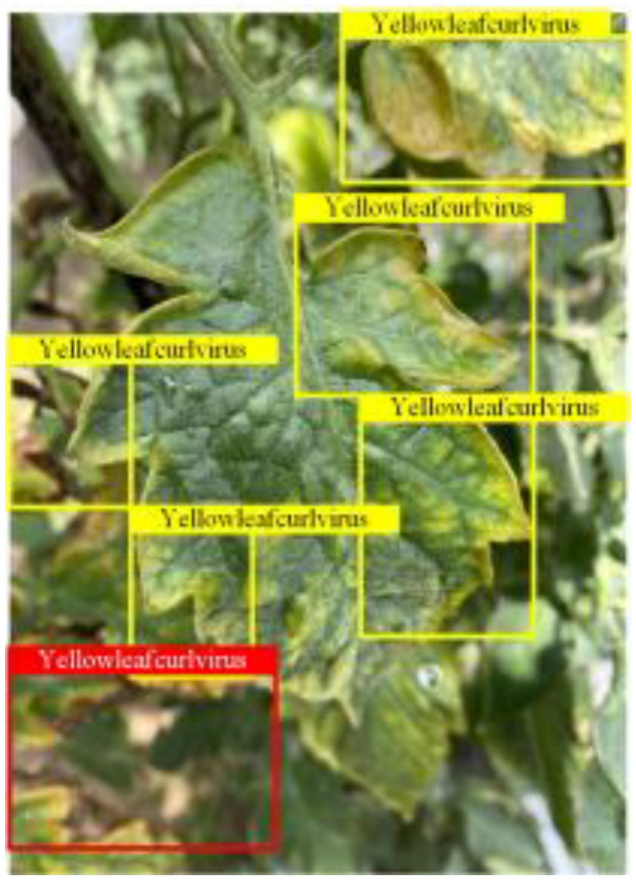	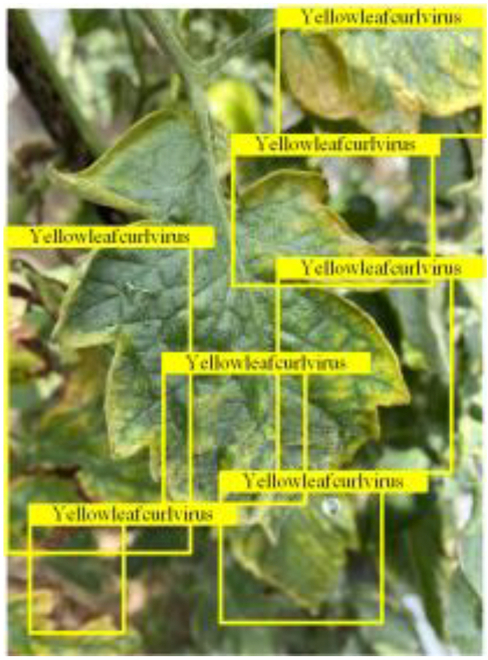	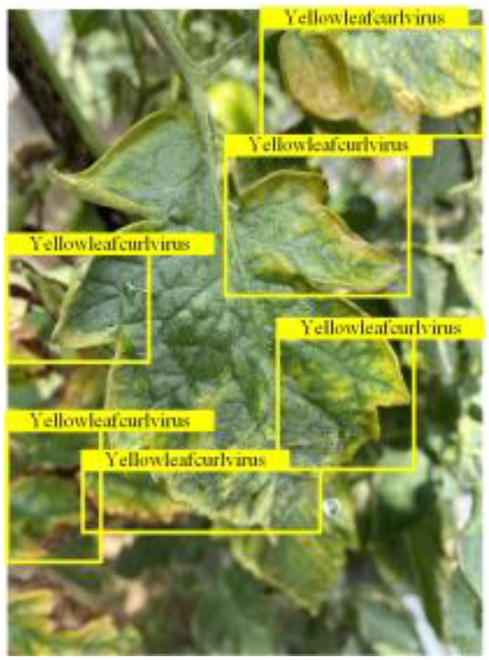	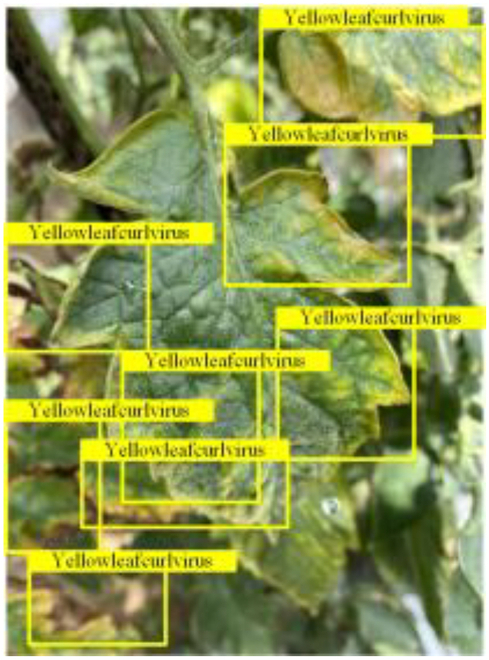	F

In the results of groups A and E, we noticed that PDC-VLD not only recognized more small diseases than VLDet but also had a better detection effect on a wider disease coverage area.

In group B, representative images with background interference near the diseased area were selected. The detection results show that VLDet tends to incorrectly, yet confidently, misidentify part of the background as the disease itself. In contrast, PVT-C and DINO showed higher global discrimination ability in distinguishing the disease from the soil background.

In groups C, D, and F, we focused on the ability of the network models in generating candidate detection frame localization. From the results, DINO showed an advantage in reducing the number of duplicate detection frames generated in the overlapping areas of the spots, while both PVT-C and DINO successfully reduced the generation of erroneous detection frames.

Overall, PDC-VLD outperforms the VLDet in terms of overall performance, not only in terms of accuracy but also in terms of optimizing the reduction of duplicate detections and false detections.

### Experiment comparing PDC-VLD with other models

To further analyze the performance of PDC-VLD, we conducted comparative experiments with several traditional and current state-of-the-art open-vocabulary target detection methods on the same test environment and test set. Table 7 shows the test results. It can be seen that our model has a substantial improvement in all categories, with a 10.2% improvement in ${\mathrm{mAP}}_{\text{novel}}^{50}$, and 15.8% and 14.4% in ${\mathrm{mAP}}_{\text{base}}^{50}$and ${\mathrm{mAP}}_{\mathrm{all}}^{50}$, respectively. This shows the superiority of our network.

**Table 7.** Experiment comparing PDC-VLD with other models

Method	${\mathrm{mAP}}_{\text{novel}}^{75}$	${\mathrm{mAP}}_{\text{novel}}^{50}$	${\mathrm{mAP}}_{\text{base}}^{50}$	${\mathrm{mAP}}_{\mathrm{all}}^{50}$	AR
OVR-CNN [23] (CVPR’21)	32.1	40.4	68.0	61.1	33.1
RegionCLIP [25] (ICLR’22)	34.3	44.7	76.2	68.3	35.6
Detic [32] (ECCV’22)	33.2	45.4	68.4	62.6	28.5
PB-OVD [28] (ECCV’22)	37.1	48.7	67.3	62.6	32.8
HierKD [27] (CVPR’22)	29.7	38.1	72.2	63.6	35.0
LocOV [31] (GCPR’22)	33.5	46.4	72.3	65.8	30.1
OV-DETR [33] (ECCV’22)	39.6	47.1	73.1	66.6	38.2
PromptDet [30] (ECCV’22)	39.3	50.4	50.9	50.7	23.1
OADP [34] (CVPR’23)	40.1	48.9	74.4	68.0	37.4
VLDet [35] (ICLR’23)	44.5	51.0	71.9	66.6	38.1
Our	56.4	61.2	87.7	81.0	45.5

As can be seen from the table, our benchmark model, VLDet, although not the best on ${\mathrm{mAP}}_{\text{base}}^{50}$, has achieved good results on ${\mathrm{mAP}}_{\text{novel}}^{50}$, which is our main judging metric. This is also one of the reasons why we chose it as our benchmark model.

Compared to the other models in the table, PDC-VLD is the most suitable model for tomato leaf disease detection. To elucidate the reasons why PDC-VLD outperforms the other models in terms of performance, we made the following speculations:

a. PDC-VLD is based on the VLDet framework, which has been shown to perform best in recognizing new categories, a core metric for evaluating model performance.

b. By adopting PVT-C as the feature extractor, PDC-VLD has enhanced the network’s ability to integrate features, thereby improving the model’s accuracy in distinguishing the boundaries between tomato leaf diseases and complex backgrounds. The introduction of PVT-C provides advanced feature extraction capabilities, particularly when dealing with natural images that have complex textures and colors, aiding the model in learning more complex feature representations and increasing sensitivity to subtle differences.

c. In the PDC-VLD model, we have introduced a tomato leaf disease anchor box calibration strategy based on DINO, marking the first of its kind within the VLDet framework. This approach has effectively addressed the issue of repetitive predictions by the model. The strategy plays a pivotal role in eliminating redundant and incorrect predictions generated by the model, substantially reducing the likelihood of false detections. Consequently, this enhances the accuracy and reliability of the final prediction outcomes.

d. In this study, we have incorporated CFG into the PDC-VLD model, and we utilize a pre-trained lightweight CNN model to extract key features from the hidden layers of the network. This step substantially simplifies the complexity of knowledge transfer and network training. With such a structural design, we have greatly enhanced the performance of tomato leaf disease detection.

e. In this paper, we have meticulously curated a homemade dataset, strictly excluding any images that were blurry or of low quality to ensure the highest level of image clarity. By focusing on high-quality, clear images, we have provided a more effective and efficient learning process for our model. This method not only enhances the model’s ability to accurately interpret and analyze data but also ensures that training is conducted under optimal conditions, thereby minimizing the noise and errors that could arise from poor-quality inputs. Consequently, this careful planning of the dataset is critical for optimizing the performance of the model, leading to more reliable and precise outcomes in its application.

### Generalization experiments

In order to verify the model’s adaptability to different plant disease datasets, we incorporated 2 corn leaf disease images not found in the training data; these images were obtained from[57] , including northern leaf blight and gray leaf spot, a total of 796 images. Each image was paired with a corresponding text description, which was obtained from the Internet. Based on the original tomato leaf disease model, we tested the model’s ability to recognize new disease types by replacing class embeddings and word vector groups to cope with the disease types presented by corn leaves. The experimental and visualization results are shown in Table 8, respectively. The results show that our model has a good cross-domain generalization ability and can be migrated from the disease recognition task of one crop to another, which lays the foundation for future applications on other plant diseases.

**Table 8.** Experiments migrated to a maize dataset

Method	${\mathrm{mAP}}_{\text{novel}}^{50}$	Gray leaf spot	Northern Leaf Bligh
VLDet	24.6	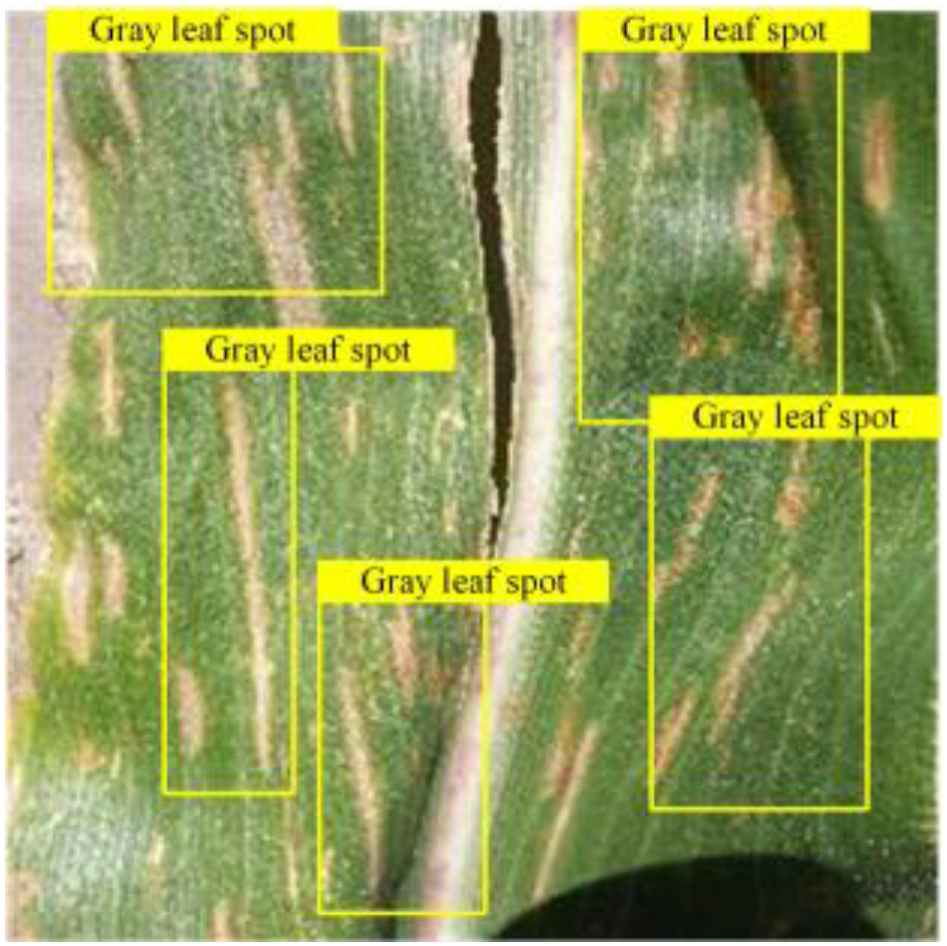	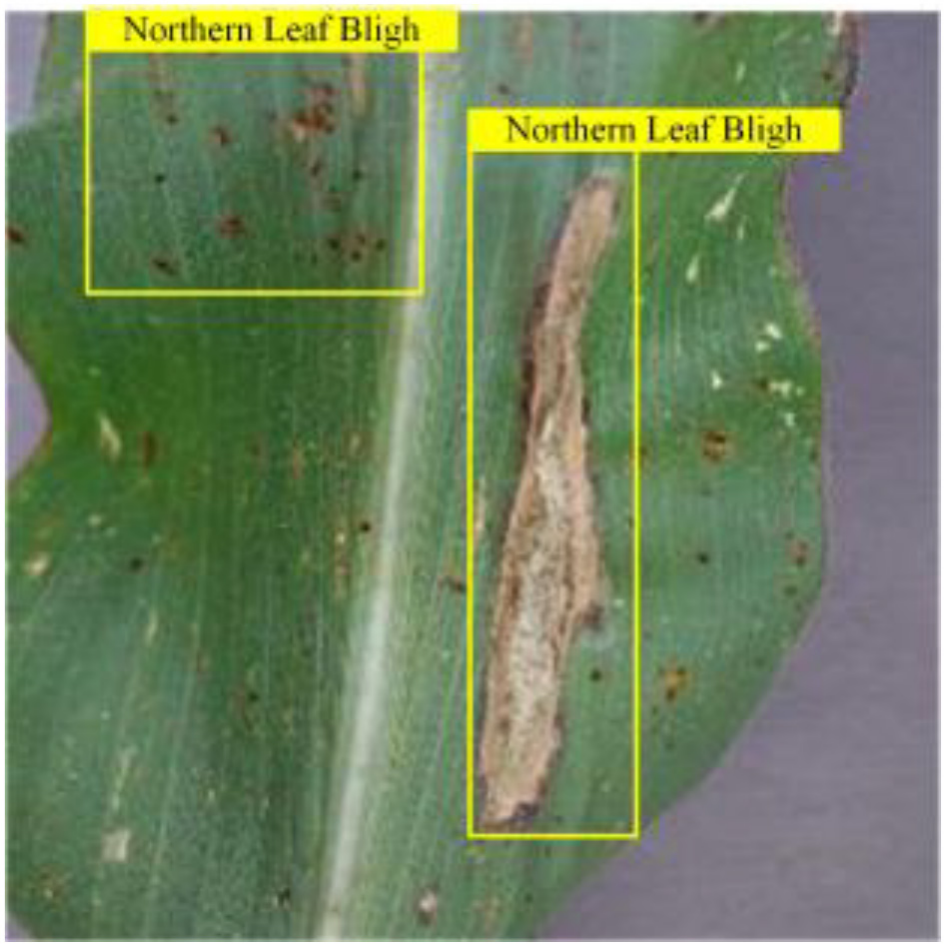
Our	36.3	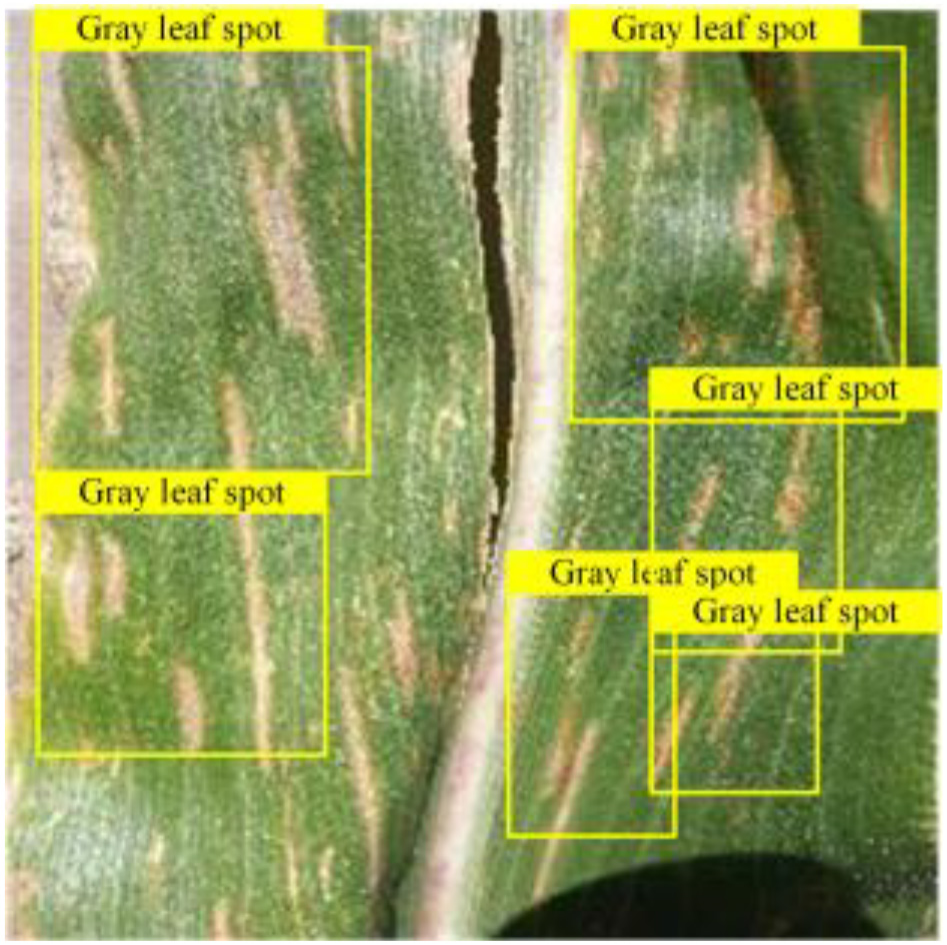	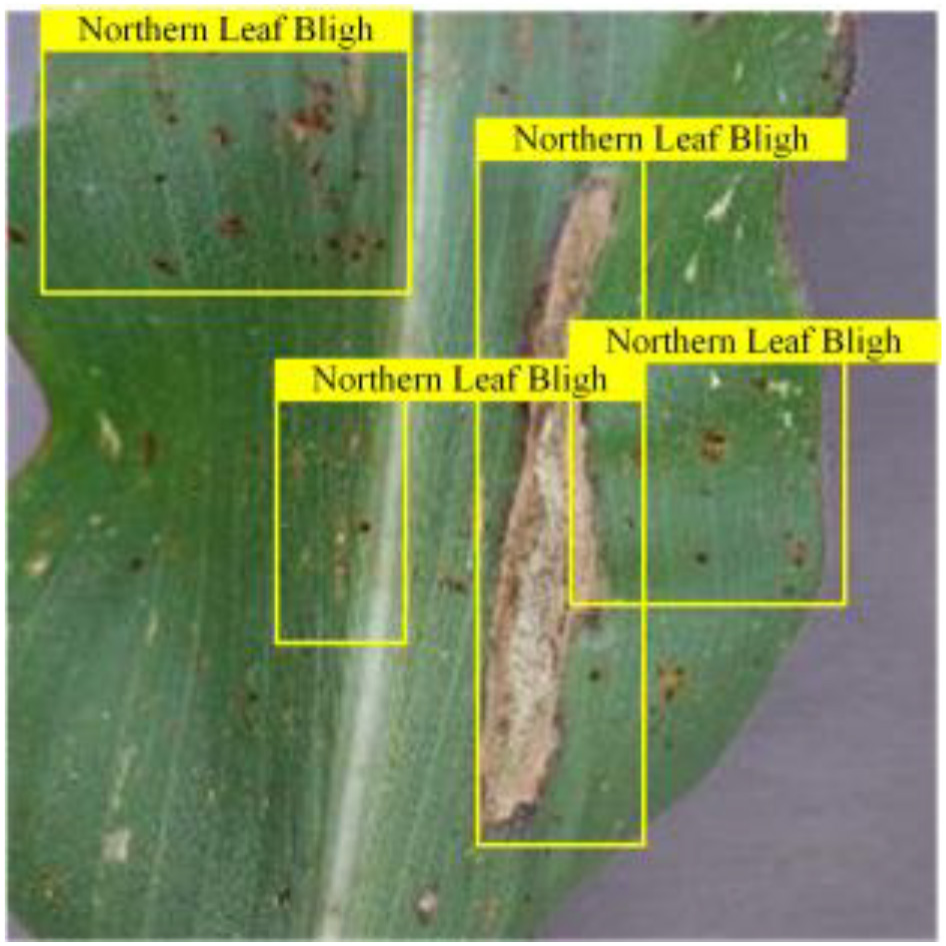

### Rationality experiment of dataset design

To evaluate the impact of dataset design on PDC-VLD, this study conducted an in-depth analysis of the differences in dataset construction strategies. Specifically, plant diseases with varying word counts in their names—early blight, septoria leaf spot, and yellow leaf curl virus—were chosen as case studies to explore the potential impact of template design on classification performance. Moreover, the study was further divided into 2 scenarios: one where the dataset explicitly contained detailed disease category information (condition A), and another where such information was absent (condition B). Additionally, a comparison was made between the use of templates (condition C) and not using templates (condition D) to determine their specific effects on classifier performance. Each disease category has 1,000 images with annotations, and each template has 2,000 text descriptions. The yellow frames indicate correct selections, while the red frames indicate incorrect ones. Through this approach, we aim to uncover the precise impact of nuanced differences in dataset design on the accuracy of PDC-VLD recognition.

As shown in Table 9, analysis of group B reveals that when disease names are omitted from the text descriptions, the network fails to recognize the disease. Observing group A + D, a trend becomes apparent: if the text deviates from the designed template and the word count exceeds 3, the network’s performance drops sharply. This is likely due to the network misinterpreting the entire phrase as an individual noun. Furthermore, we also observed that text descriptions lacking disease names could still slightly improve the network’s recognition rate. We believe that such data may provide the network with subtle global feature cues.

**Table 9.** Rationality experiment of dataset designs

Method	${\mathrm{mAP}}_{\text{novel}}^{50}$	Early blight	Septoria leaf spot	Yellow leaf curl virus
A + C	61.1	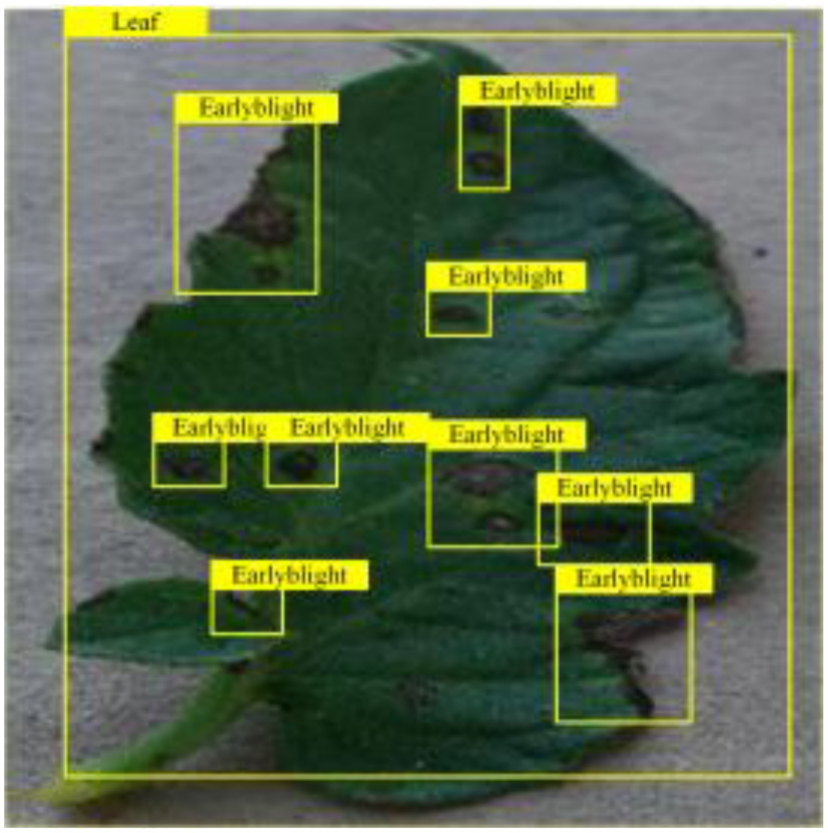	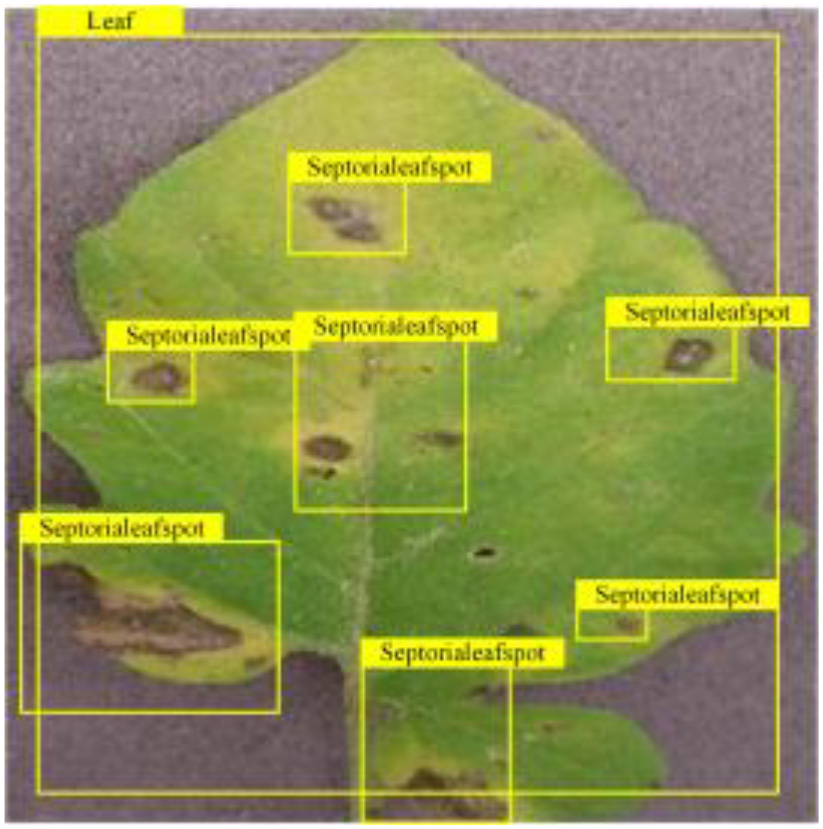	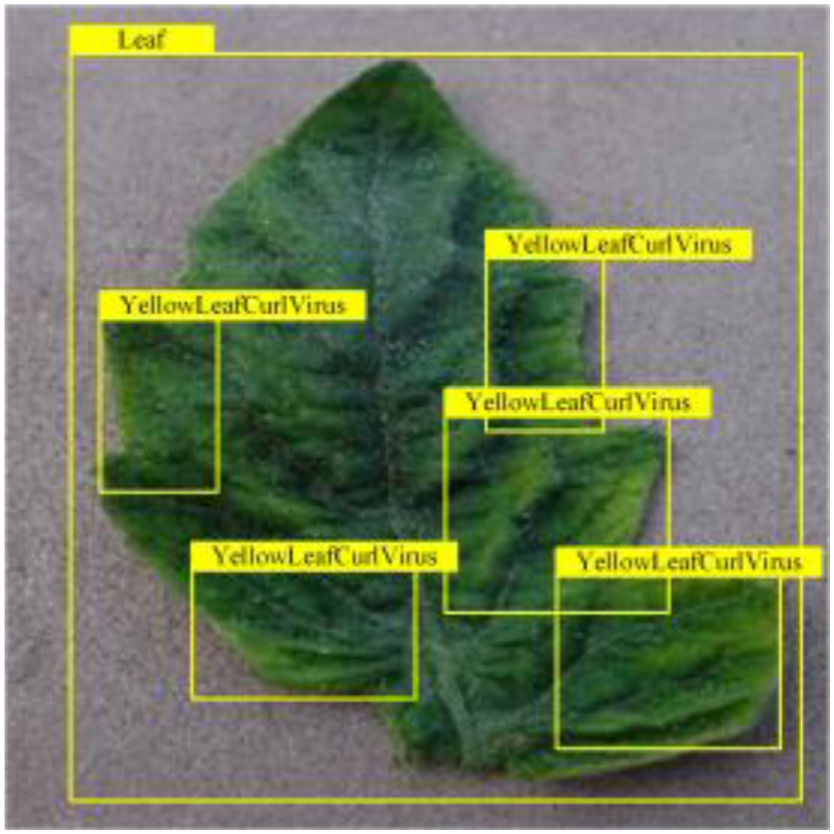
A + D	28.7	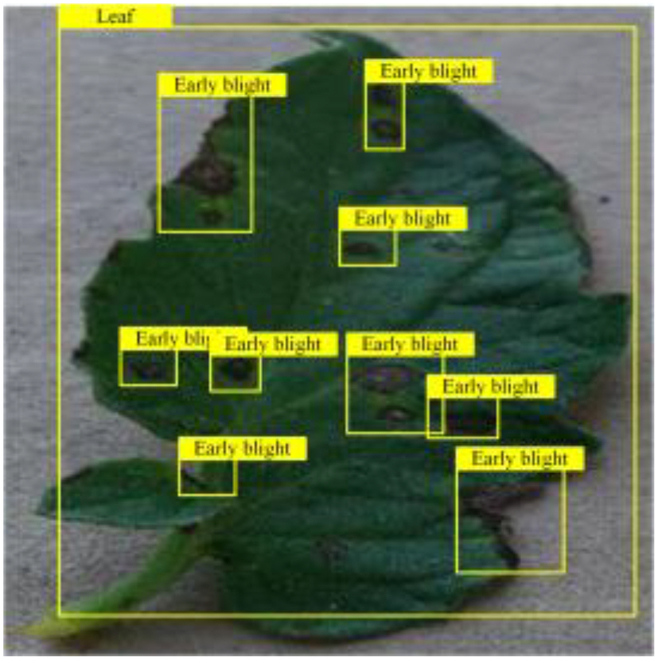	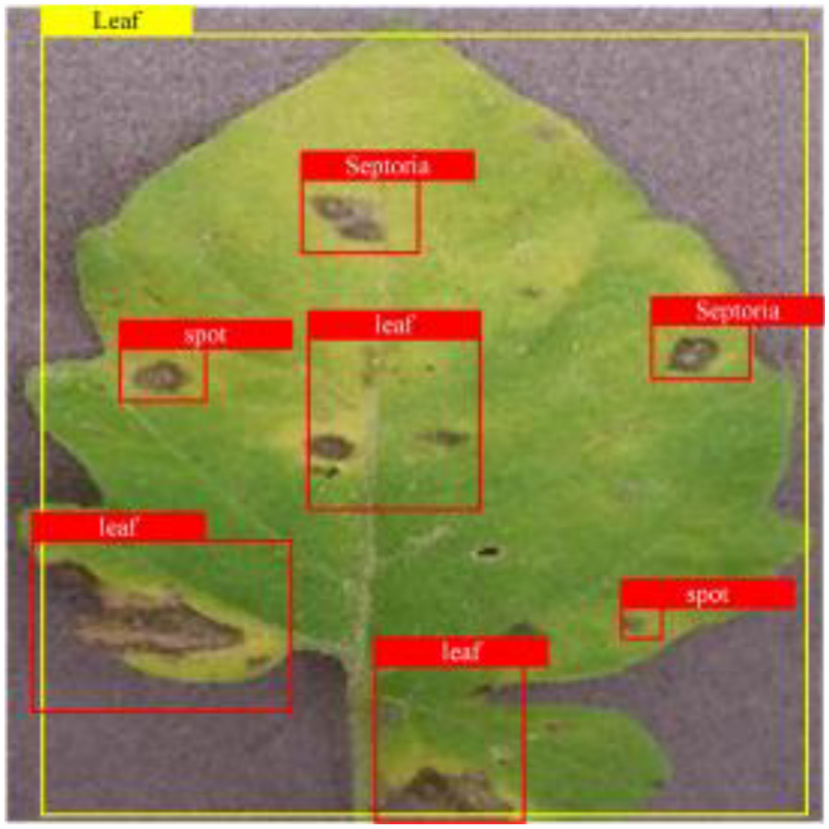	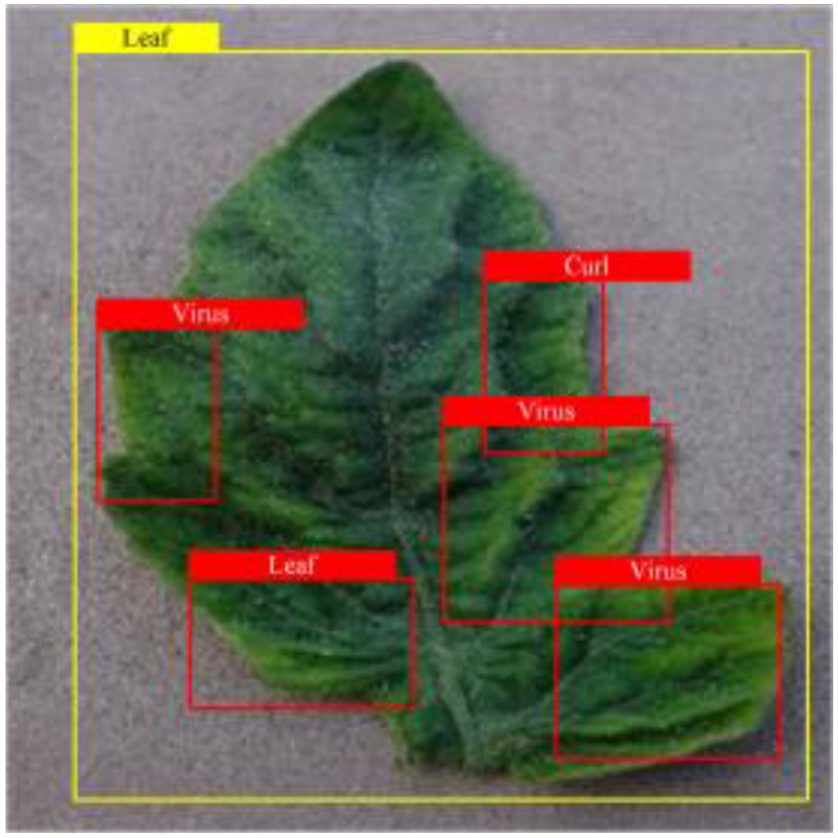
B	1.1	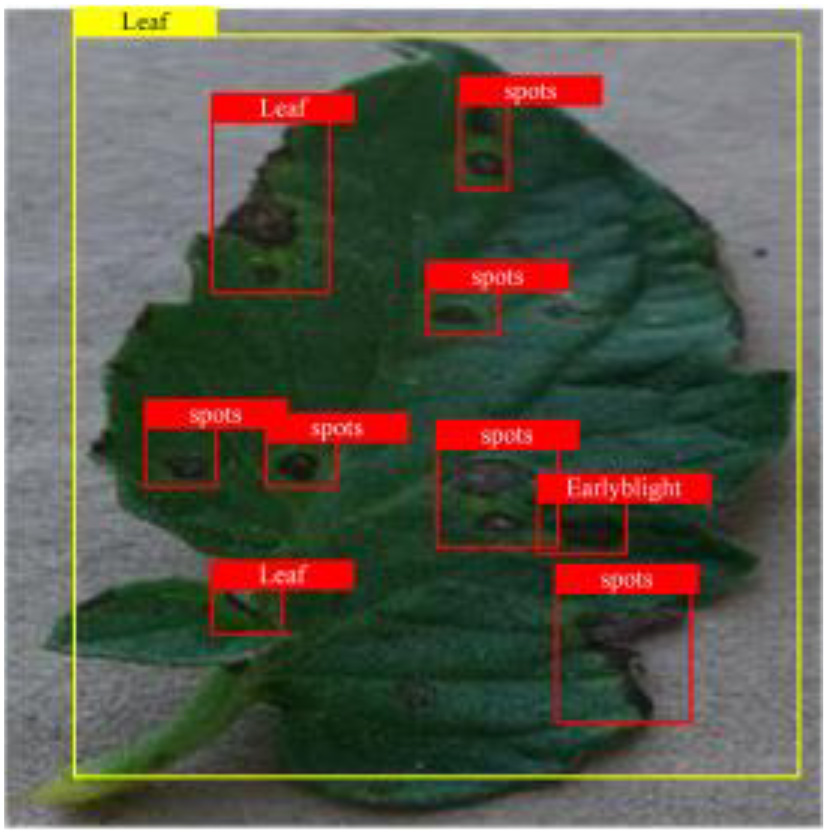	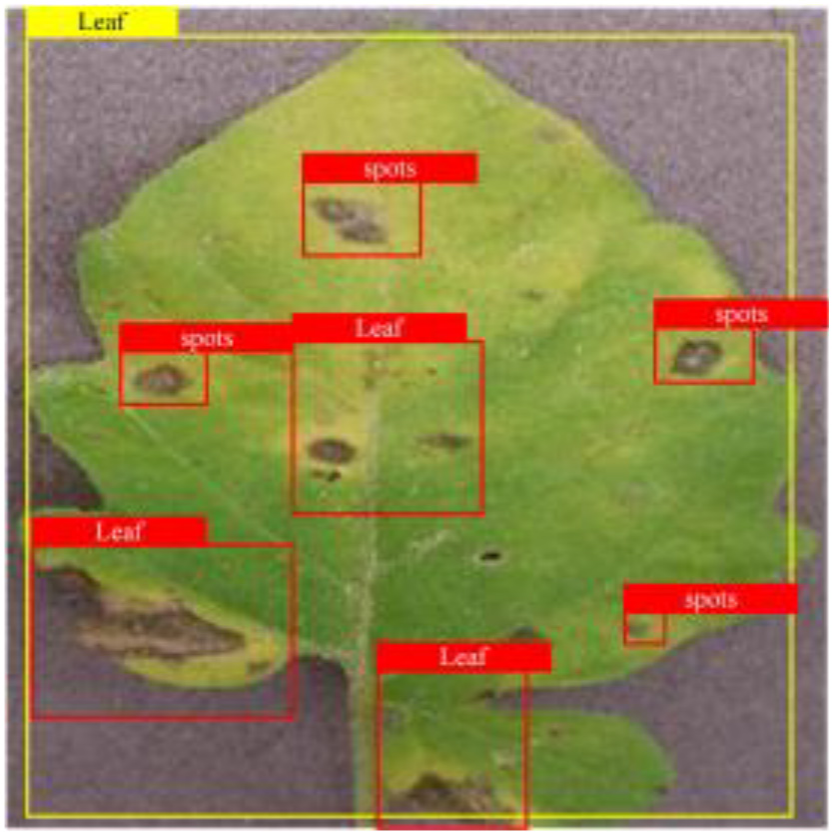	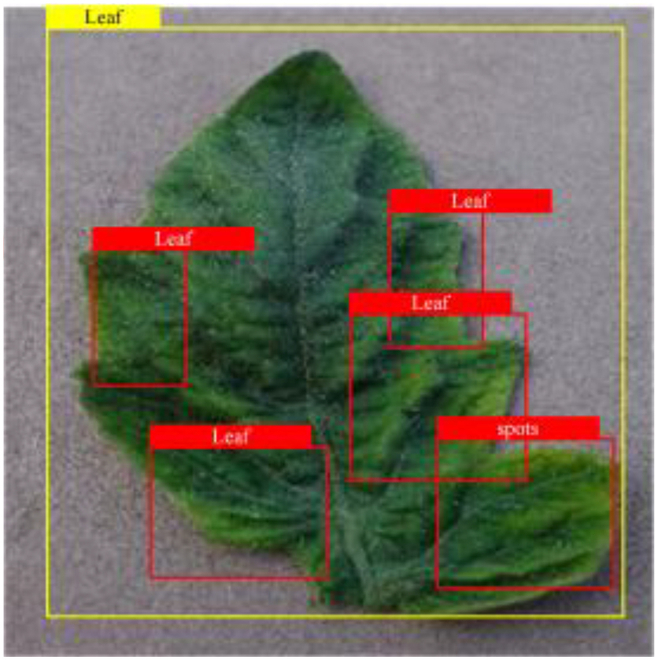
A + B + C	61.2	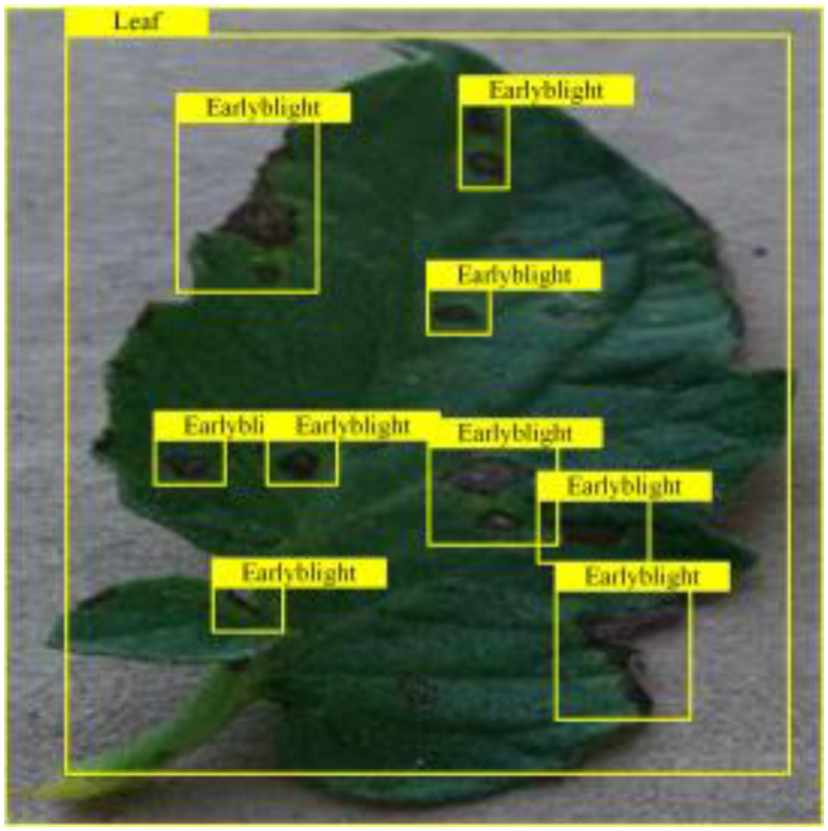	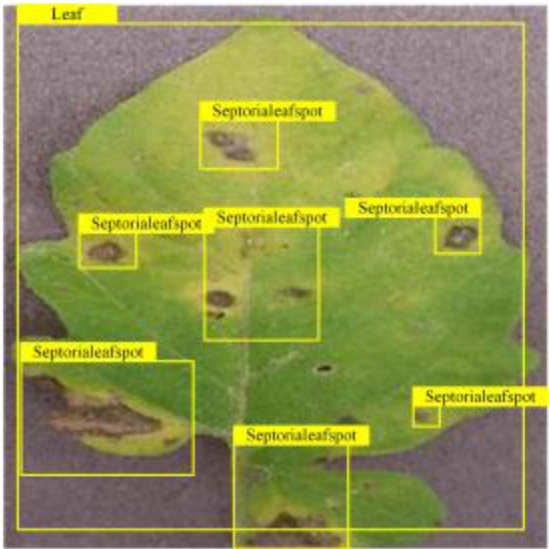	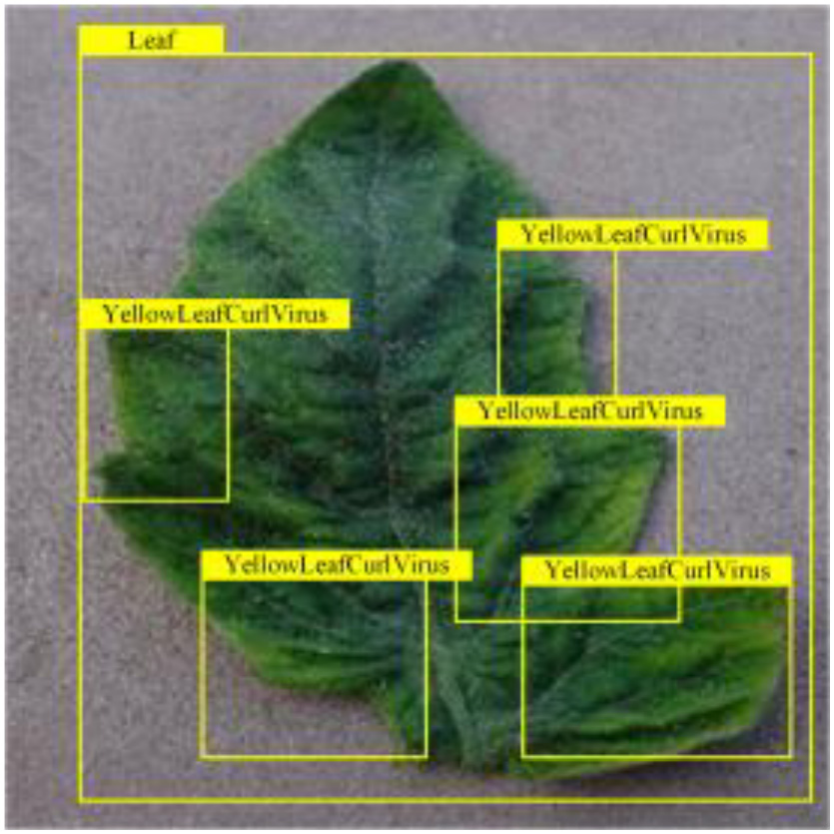

### Real-world experiments

In this section, we designed various types of noise and lighting conditions to test the model’s performance in real-world scenarios. We divided the experiments into 2 groups: noise and background. In the noise group, we applied Gaussian noise at levels 0.03 and 0.1, as well as salt and pepper noise at levels 0.03 and 0.1. In the background group, we used grayscale images, low light, and strong light conditions to conduct the experiments.

Through these experiments, we aim to thoroughly evaluate the model’s performance under various complex conditions to verify its robustness and effectiveness in practical applications (Table 10).

**Table 10.** Real-world experiments

Method	No noise	Gaussian noise		Salt-and-Pepper noise	
		0.03	0.1	0.03	0.1
VLDet	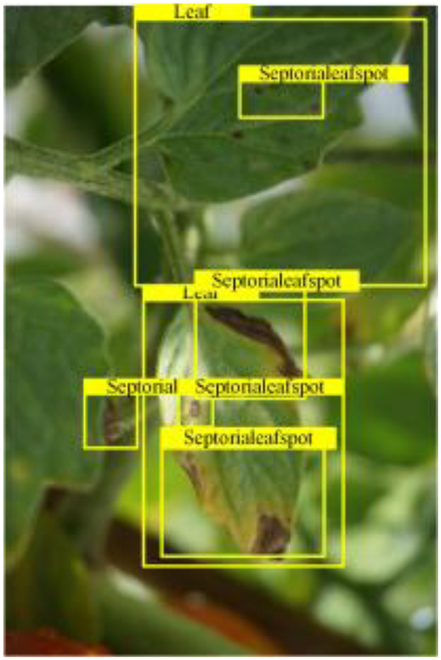	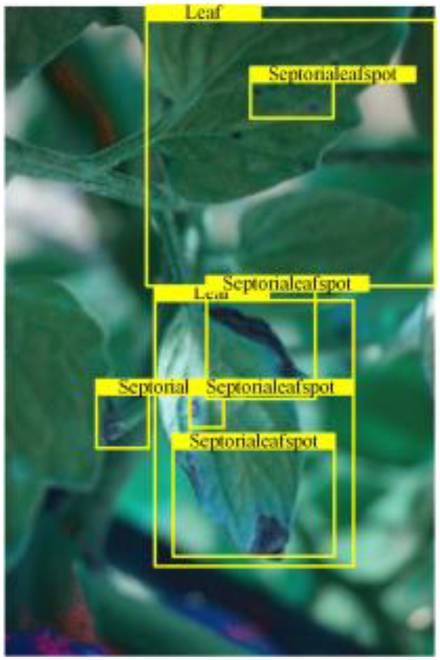	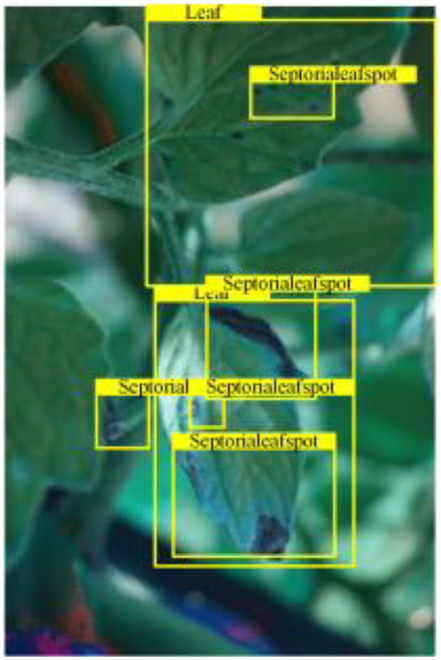	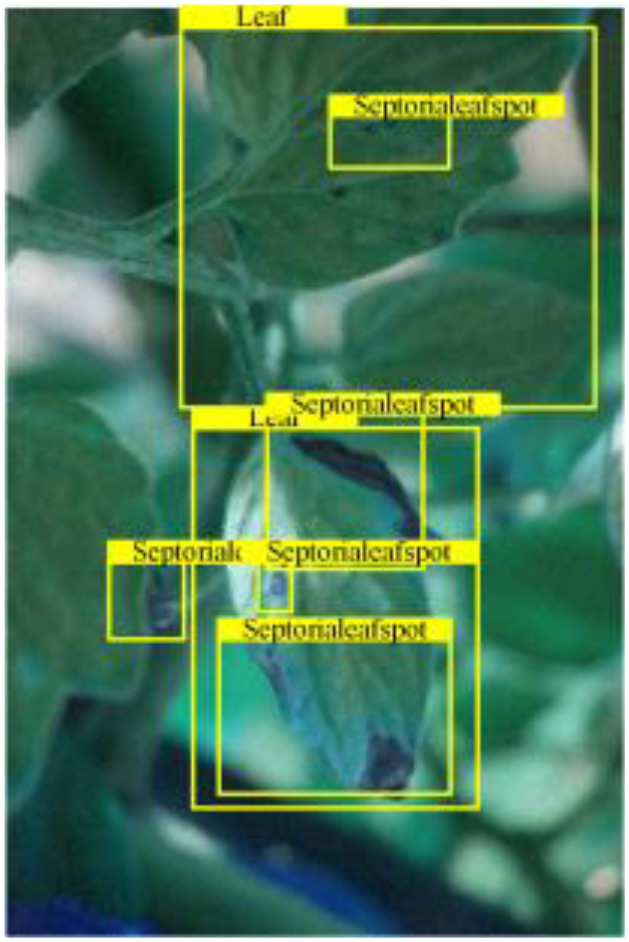	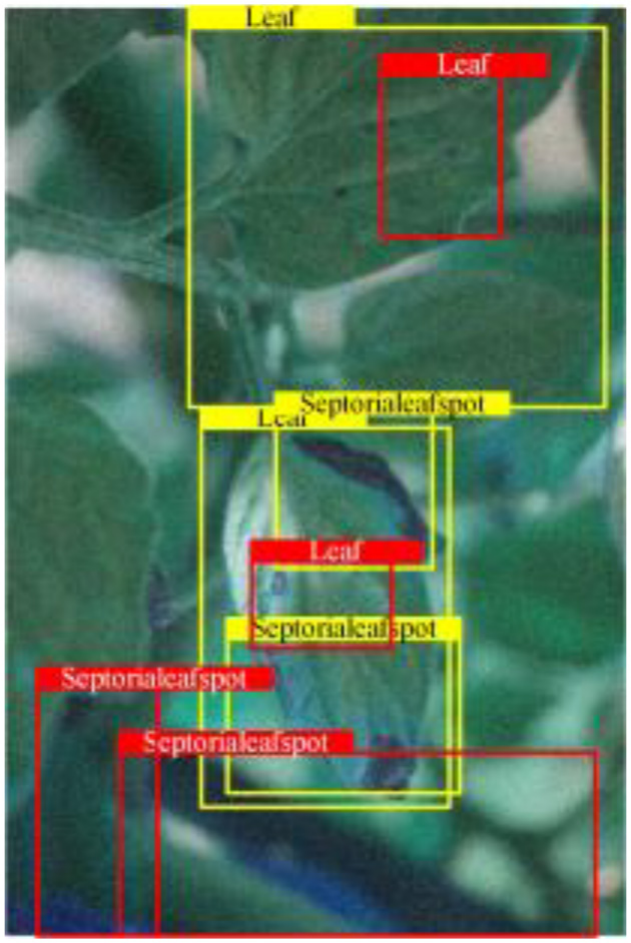
PDC-VLD	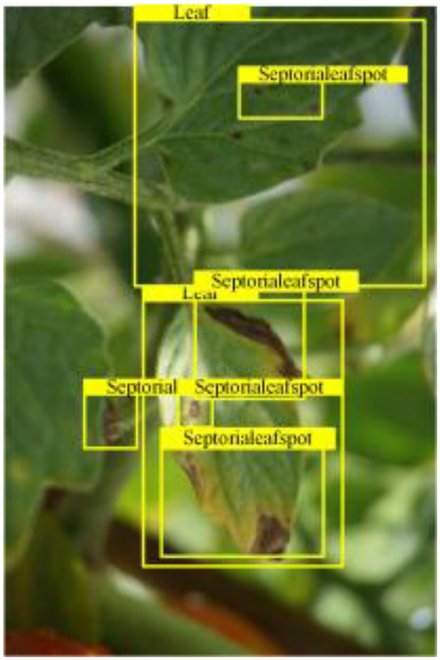	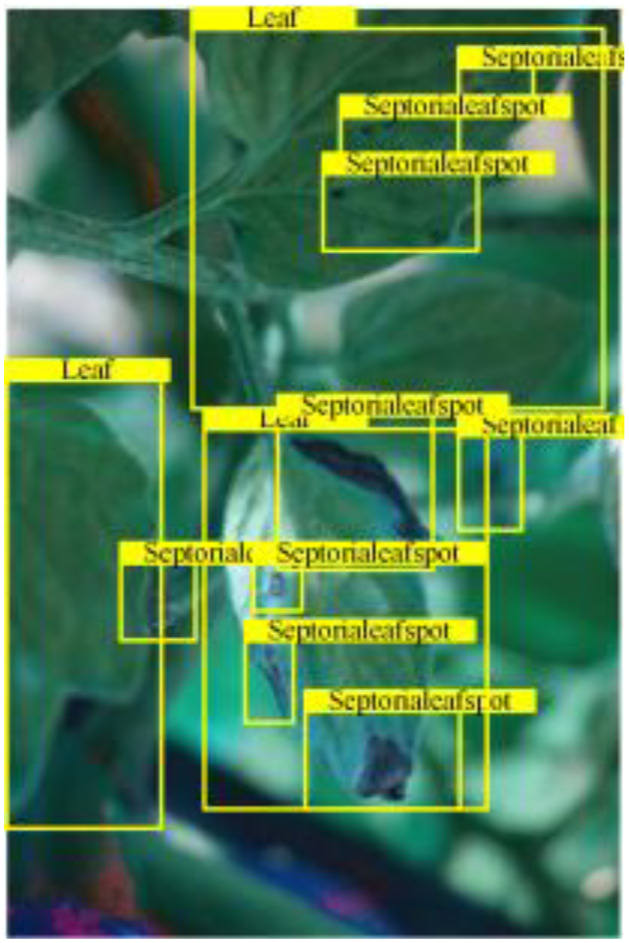	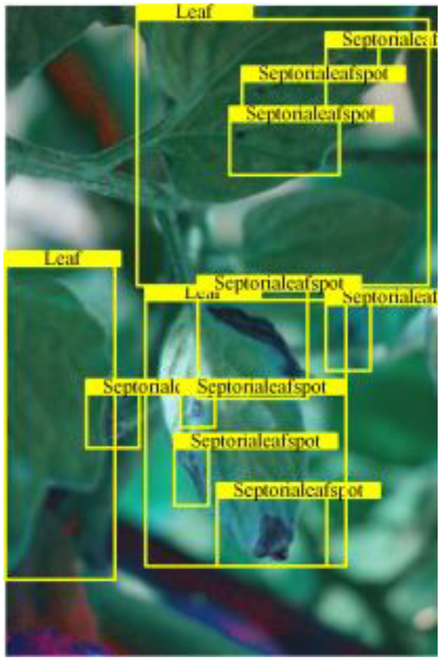	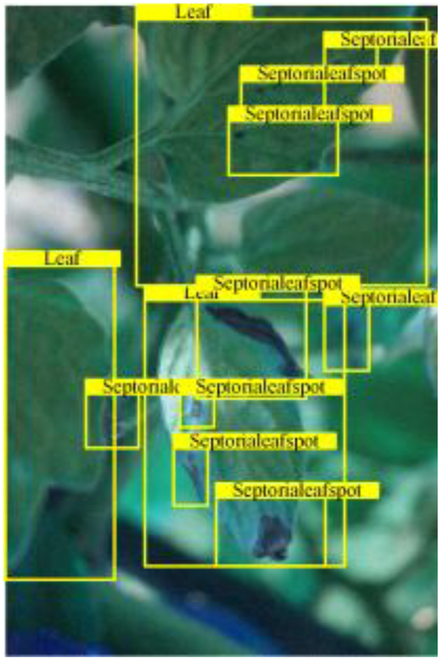	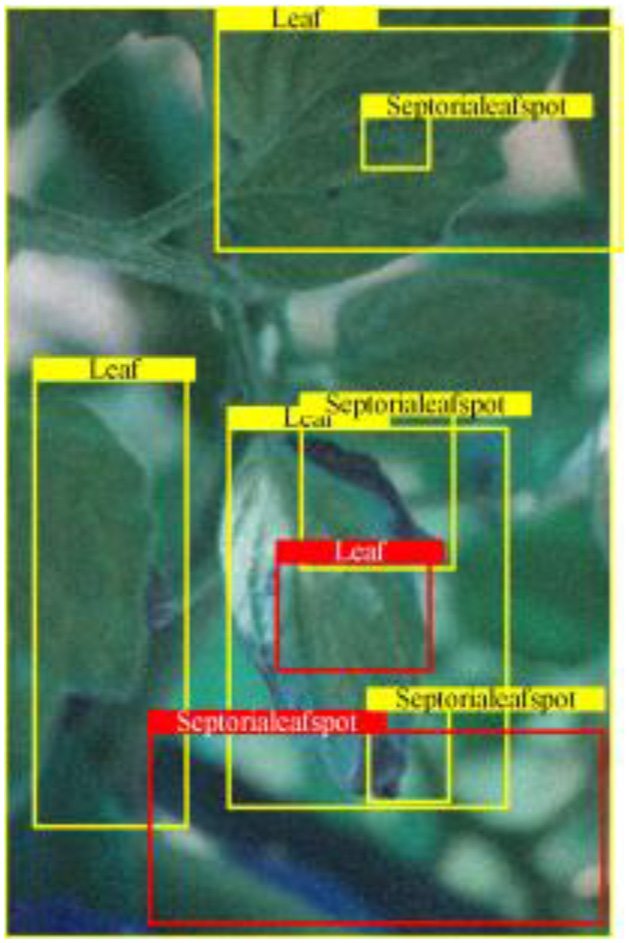
	**Normal intensity**	**Grayscale**	**Low light**	**Strong light**	
		**0.4**	**0.7**	**2**	**3**
VLDet	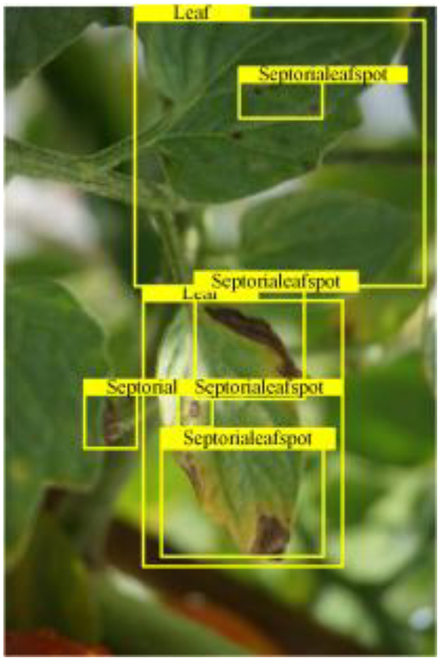	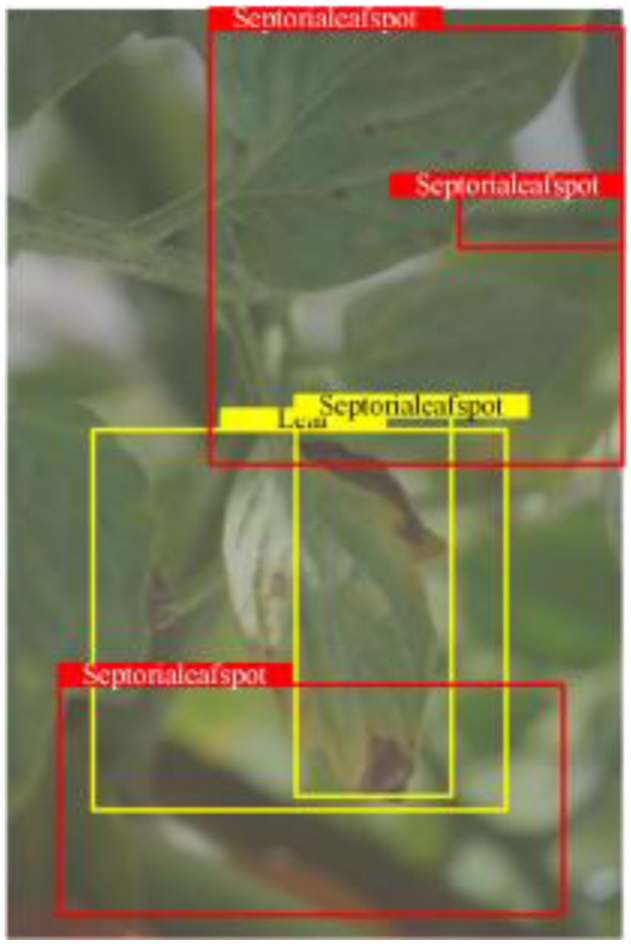	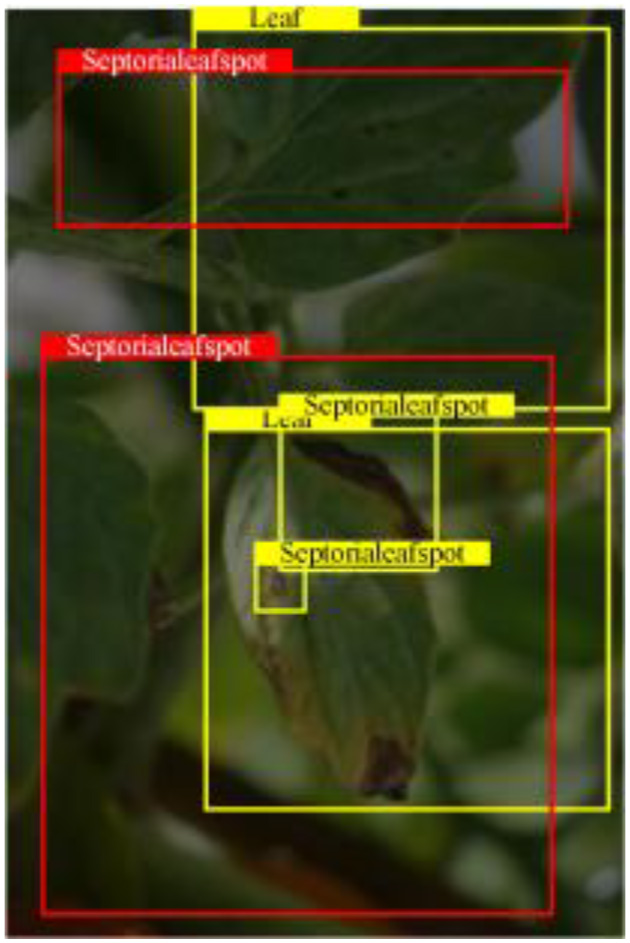	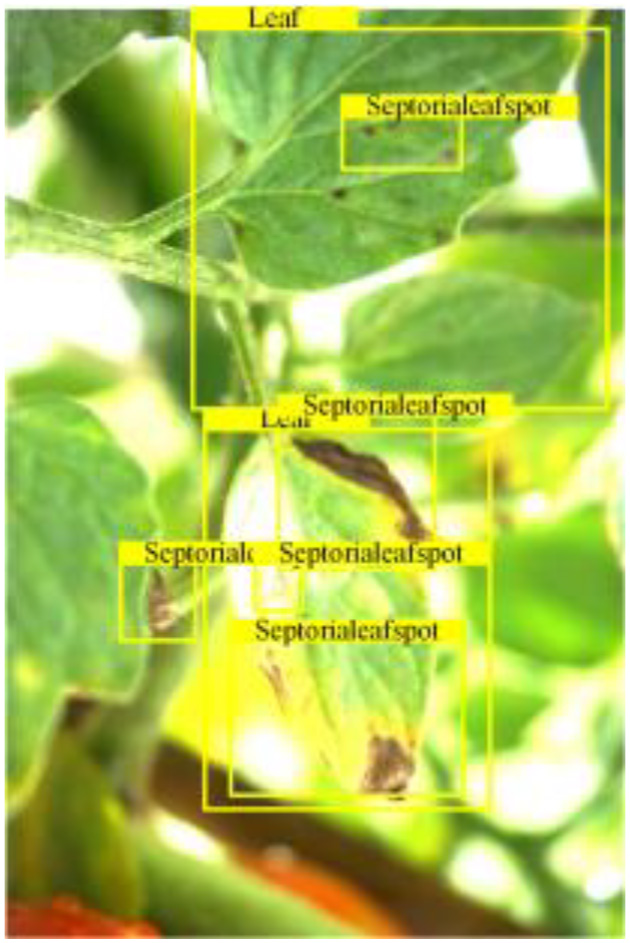	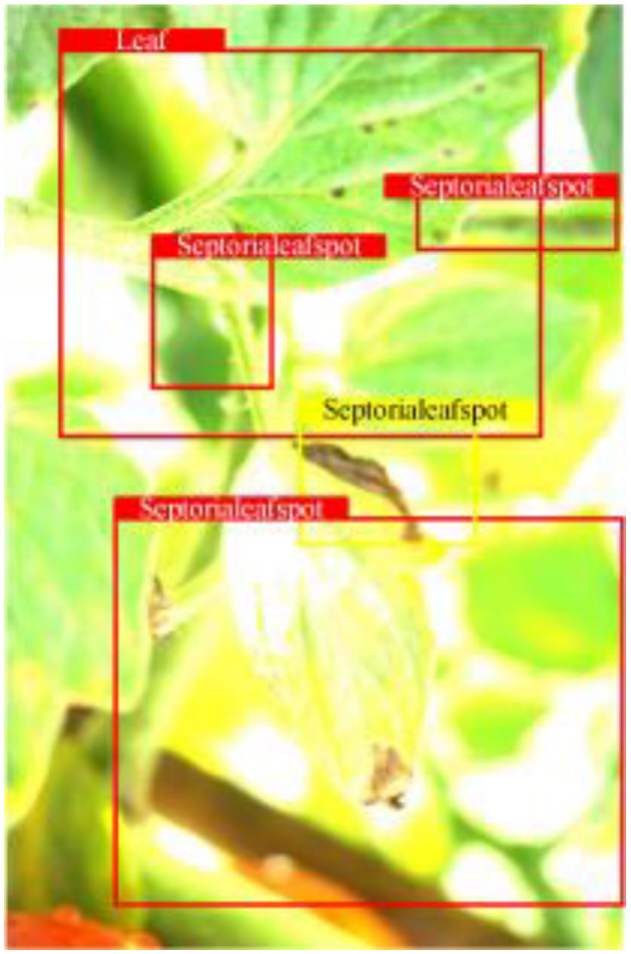
PDC-VLD	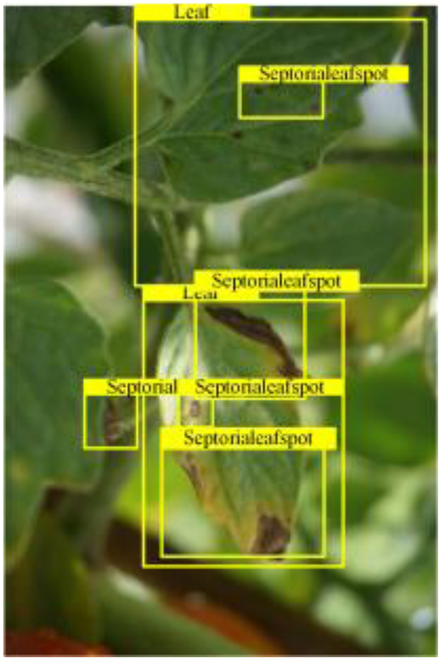	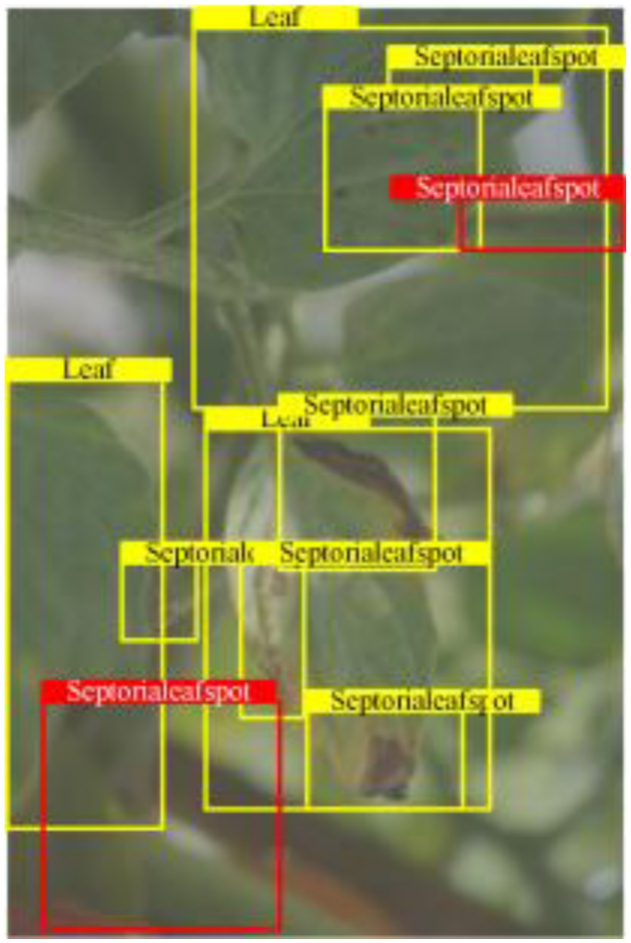	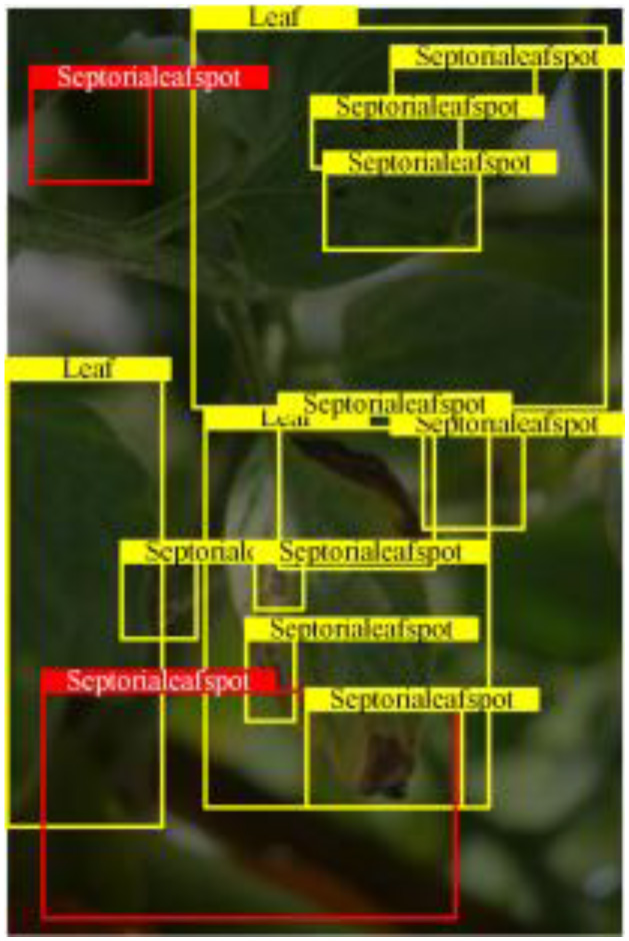	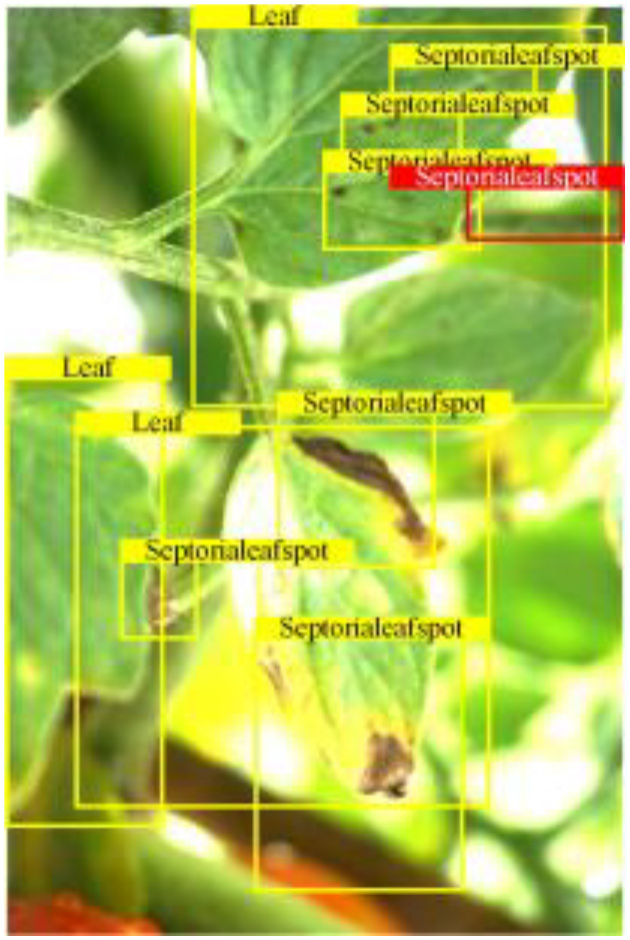	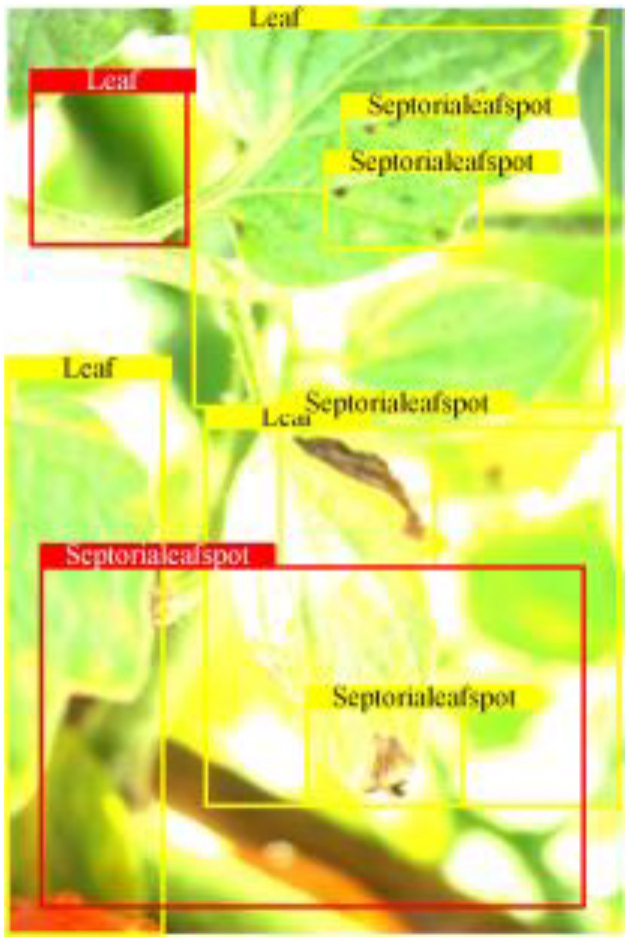

It is evident that our model can maintain a certain level of accuracy even in the presence of high noise, which is higher than that of the baseline network. This is due to the fact that the modules we have improved have effectively increased the recognition rate with clean datasets. Additionally, our model outperforms the benchmark model under grayscale, low light, and strong light conditions. These all demonstrate the superiority of our model.

## Discussion

**Fig. 4.**
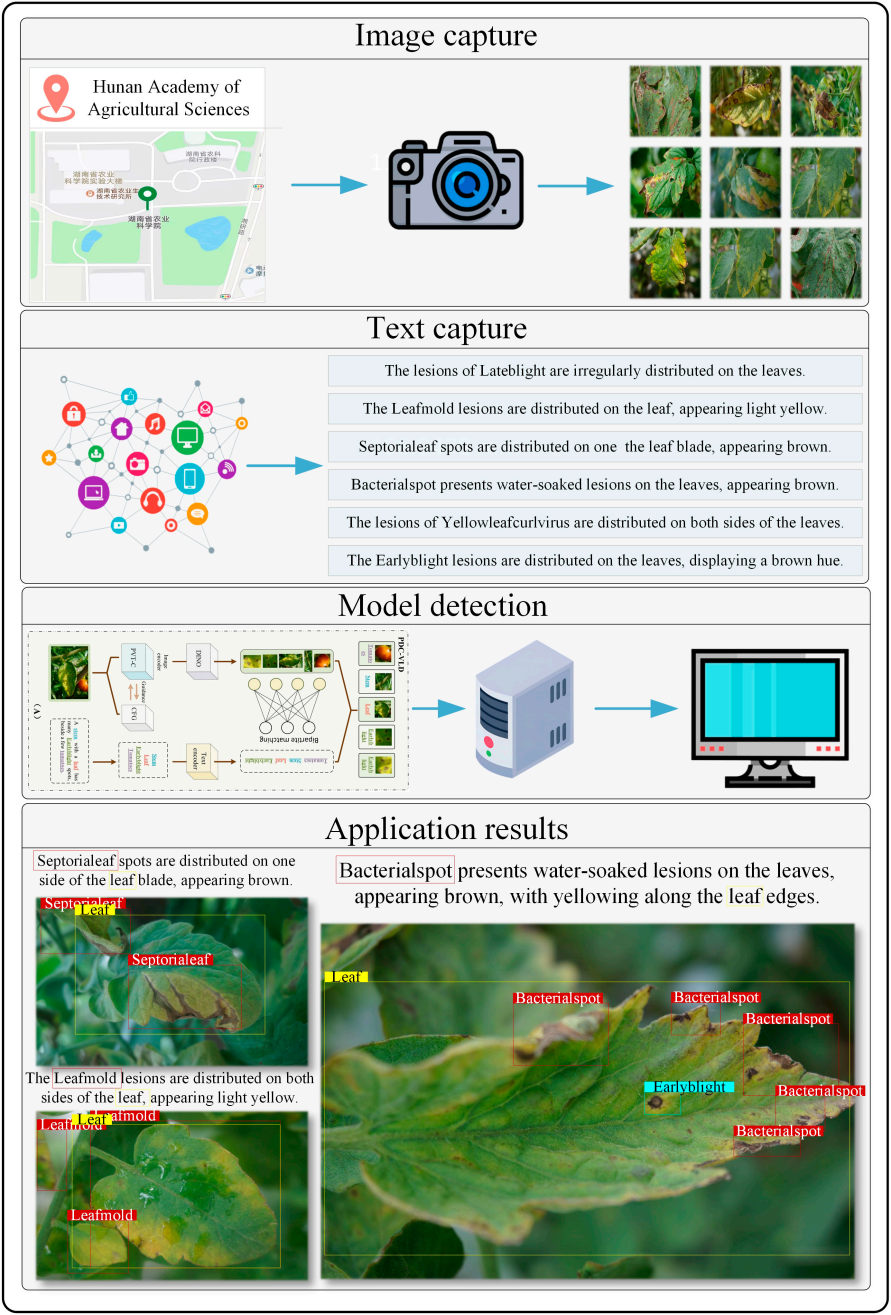
Schematic diagram of the PDC-VLD-based disease assessment system.

To evaluate the performance of the PDC-VLD model for target detection of tomato leaf diseases, we built an automated detection system, the architecture of which is demonstrated in Fig. 4. Initially, we collected 400 images depicting 4 tomato leaf diseases (3 of the new categories and one of the base categories for generalizability experiments through field photography in Yueyang, Hunan, China. We then used Internet-generated text to create annotation labels for the images. Next, the input image size is automatically converted to 256 × 256 in order to match the model. Finally, the results of the input images and the target detection are displayed via a terminal. After the deployment is completed, the tomato disease leaf images are sent over the network to the server for target detection, and the detected disease results are fed back in a timely manner to assist in the specific analysis and evaluation of tomato diseases. Figure 4 shows some of the outputs of the detection system. The highlighted area indicates the identification of the error or disease. When different diseases exhibit similar features at various stages, such incidents particularly occur. This indicates that the model needs to strengthen its target detection ability for similar diseases at the fine-grained stage. Although these occasional misidentifications do not impair the model’s judgment for target detection of the same disease, the issue remains a substantial challenge that we face. Overall, the results aligned with expectations. The model demonstrated efficacy in identifying tomato leaf diseases, and its proficiency in recognizing novel diseases was generally satisfactory.

Looking forward, to ameliorate the potential misdiagnosis due to the similarity in disease characteristics across different stages of various diseases, we are contemplating the development of an advanced open-vocabulary target detection framework tailored for fine-grained recognition. Upon the refinement and successful implementation of our framework, capable of discerning between similar fine-grained pathological features of distinct diseases, it is anticipated to serve as a more effective open-vocabulary tool for the prevention and early detection of foliar diseases. This would signify a technological advancement in precision control for the management and treatment of leaf diseases.

Furthermore, to facilitate field deployment in agricultural settings, we are considering the integration of Internet of Things (IoT) sensor technology with the enhanced open-vocabulary target detection methodology. Such augmented detection technology is projected to allow for more accurate identification of the various stages of leaf disease progression. This broader approach is expected to strengthen comprehensive disease surveillance, enabling the system to detect foliar diseases across different stages, thereby allowing for timely and targeted interventions to effectively manage and control the spread of diseases.

## Conclusion

In this study, we successfully pioneered the application of OVD technology to the identification of tomato leaf diseases. We developed a multimodal model, PDC-VLD, based on OVD techniques. This model accurately identifies novel tomato leaf diseases through image–text pairs without the need for manual annotations, substantially reducing the human resource investment required to recognize new categories. We trained and tested our model on a self-constructed tomato leaf disease image dataset, achieving excellent results. Specific conclusions are presented as follows: Ablation experiments show that PVT-C, DINO, and CFG have better effects on the detection of tomato leaf diseases, where ${\mathrm{mAP}}_{\text{novel}}^{50}$ increased by +4.7%, +4.1%, and +5.5%, respectively. Under the same experimental setup, compared with VLDet, PDC-VLD improved ${\mathrm{mAP}}_{\text{novel}}^{75}$, ${\mathrm{mAP}}_{\text{novel}}^{50}$, ${\mathrm{mAP}}_{\text{base}}^{50}$, ${\mathrm{mAP}}_{\mathrm{all}}^{50}$, and AR by 11.9%, 10.2%, 15.8%, 14.4%, and 7.4%, respectively. Compared to the current mainstream OVD networks, our PDC-VLD model demonstrates superior performance across several key metrics. Specifically, it achieves ${\mathrm{mAP}}_{\text{novel}}^{75}$ of 56.4%, ${\mathrm{mAP}}_{\text{novel}}^{50}$ of 61.2%, ${\mathrm{mAP}}_{\text{base}}^{50}$ of 87.7%, ${\mathrm{mAP}}_{\mathrm{all}}^{50}$ of 81.0%, and AR of 45.5%, all of which surpass existing models. These results indicate that PDC-VLD is effective in handling the encoding of novel category images and the identification of disease. However, we also recognize that there are still many challenges that need to be further addressed: (a) High dependence on image encoders: The current VLDet framework exhibits a substantial reliance on image encoders, which results in high resource demands. Future efforts will explore strategies to reduce this dependency on high-resource encoders, potentially through optimizing encoder designs or seeking alternative solutions with lower resource requirements. (b) Bias between visual and linguistic data: The existing design has not adequately addressed the issue of bias between visual and linguistic data. Future research will focus on identifying and mitigating these biases, developing methods to handle the discrepancies introduced by different data sources.

In summary, our research has initially validated the potential application of OVD technology in the identification of tomato leaf diseases. This novel deep learning framework not only advances the theory and practice of agricultural disease diagnosis but also opens new avenues for the automation and precise detection of crop diseases. We anticipate continuing to optimize the model and extending its application to a broader range of crops and disease detection scenarios, aiming for enhanced performance and assessing its potential impact on agricultural production and economic value. Despite the challenges, we believe that sustained efforts and research will help transform these challenges into driving forces for advancing the field.

## Data Availability

All datasets that were used and analyzed in this study have been uploaded to the website https://github.com/ZhouGuoXiong/PDC-VLD. Furthermore, for access to all bespoke datasets used in this study (comprising a total of 6,923 images and 13,864 texts), please contact the corresponding author.
